# A computational modeling of invadopodia protrusion into an extracellular matrix fiber network

**DOI:** 10.1038/s41598-022-05224-9

**Published:** 2022-01-24

**Authors:** Min-Cheol Kim, Ran Li, Rohan Abeyaratne, Roger D. Kamm, H. Harry Asada

**Affiliations:** 1 Departments of Mechanical Engineering, Massachusetts Institute of Technology, Cambridge, MA 02139 USA; 2 Biological Engineering, Massachusetts Institute of Technology, Cambridge, MA 02139 USA; 3 Center for Systems Biology, Massachusetts General Hospital Research Institute, Boston, MA 02114 USA

**Keywords:** Mechanical engineering, Biomedical engineering, Computational biophysics

## Abstract

Invadopodia are dynamic actin-rich membrane protrusions that have been implicated in cancer cell invasion and metastasis. In addition, invasiveness of cancer cells is strongly correlated with invadopodia formation, which are observed during extravasation and colonization of metastatic cancer cells at secondary sites. However, quantitative understanding of the interaction of invadopodia with extracellular matrix (ECM) is lacking, and how invadopodia protrusion speed is associated with the frequency of protrusion-retraction cycles remains unknown. Here, we present a computational framework for the characterization of invadopodia protrusions which allows two way interactions between intracellular branched actin network and ECM fibers network. We have applied this approach to predicting the invasiveness of cancer cells by computationally knocking out actin-crosslinking molecules, such as α-actinin, filamin and fascin. The resulting simulations reveal distinct invadopodia dynamics with cycles of protrusion and retraction. Specifically, we found that (1) increasing accumulation of MT1-MMP at tips of invadopodia as the duration of protrusive phase is increased, and (2) the movement of nucleus toward the leading edge of the cell becomes unstable as duration of the retractile phase (or myosin turnover time) is longer than 1 min.

## Introduction

Cancer metastasis is a key step in cancer progression, and it is composed of multiple processes: (1) local infiltration of cancer cells into the adjacent tissue, (2) transendothelial migration of cancer cells into blood or lymphatic vessels (intravasation), (3) escaping from the immune system and survival in the circulatory system, (4) attachment of cancer cells to the vessel wall and migration out of the vessel (extravasation), and (5) subsequent proliferation in the distant organs leading to the establishment of colonies at the secondary sites^1,2^. Among these complicated processes, both intravasation and extravasation require invasive cancer cell migration into the basement membrane (BM) that is composed of highly specialized extracellular matrix (ECM). Cancer cell invasion into BM is facilitated by the dynamic movement of actin-rich cellular protrusion called invadopodia^3^. Hence, examining how cancer cells utilize actin-myosin machinery during dynamic movement of invadopodia protrusion (IP), as well as how they preferentially change their morphologies during the extravasation, will provide valuable insights into the intricacies of cancer metastasis.

The formation of invadopodia is a characteristic of highly invasive cancer cells^4^. Invadopodia are elongated ventral membrane protrusions that are composed of a variety of proteins, such as actin filaments, actin-related protein-2/3 (Arp2/3) complex, neuronal Wiskott-Aldrich syndrome protein (N-WASP), cortactin, fascin, and matrix degradation enzymes. Among them, N-WASP and cortactin are essential components that can synergistically activate Arp2/3 complex, and these proteins are upregulated in malignant cancer cells^5^. Invadopodia play a critical role in the process of extravasation by inducing basement membrane (BM) disruption through local ECM degradation. In addition, it has been reported that invadopodia is potently induced by high-density fibrillary collagen (HDFC) matrix in both cancer cell lines and primary human fibroblasts^6^. To achieve this, cancer cells need to secrete matrix metalloproteinase (MMP) family to degrade local ECM with which cellular membrane interact. Specifically, matrix type 1- metalloproteinase (MT1-MMP) accumulates at tips of invadopodia, which facilitates membrane protrusion. On the other hand, an interesting study has recently revealed a new mode of invadopodia-assisted migration that is ECM plasticity-mediated and protease-independent. This mode of migration can occur when ECM pore sizes are smaller than ~ 3 μm and ECM is sufficiently plastic, which allows cancer cells to use invadopodia protrusion to physically widen the ECM pores and create an open channel for migration^7^.

Previously, some computational approaches have made it possible to obtain novel insights into the IP dynamics. These approaches include: (1) a simple model with several invadopodia penetrating into 2D crosslinked ECM^8^; (2) a cellular automata-based model with several invadopodia penetrating into 3D ECM which can be degraded and remodeled by MT1-MMP secretion at tips of invadopodia^9^; (3) a 2D mathematical model of reaction–diffusion with a minimal set of molecular interactions between actin reorganization, ECM degradation, and MMP signaling^10^; and (4) a 3D computational model for continuous or pulsatile insertion of MT1-MMP which includes the activation of proMMP-2 by MT1-MMP, MMP-2 inactivation by tissue inhibitor of metalloproteinases-2 (TIMP-2), and the formation of ternary complex (MT1-MMP:TIMP-2:TIMP-2)^11^. While providing many new insights, these previous models simplified the IP dynamics by considering only the growth and penetration of invadopodia into a 2D ECM or a 3D ECM, but both computational domains of invadopodia and ECM were modeled as a continuum without considering the discrete nature of actin filaments and ECM fibers. Consequently, those prior models need to invoke certain assumptions about the interactions between the intracellular actin network in invadopodia and the ECM fibers, and how these interactions enable cancer cell invasion.

An invading cancer cell utilizes both mechanical and chemical interactions between 3D networks of intracellular branched actin and extracellular ECM fibers at the site of invadopodia protrusions. The current work is motivated by an experimental observation that multiple cycles of invadopodium elongation and retraction is essential for cell invasion in 3D ECM^12^. To this end, we have established a computational 3D cancer cell invasion model into a discrete ECM collagen type 1 fiber network by coupling two distinct models of the intracellular branched actin network and the ECM fiber network via an invadopodia membrane. In addition, viscoelastic behaviors in the branched actin network, actin cortex layer (ACL), force transduction layer (FTL), cellular membrane, and ECM fiber network were simulated using Kelvin-Voight model (a spring and a dashpot together in parallel). We first aim to look at 3D cancer cell invasion with three different duration times in the protrusive phase and three duration times in the retractile phase (or turnover rates of non-muscle myosin II) to characterize invadopodia protrusive shapes as well as to understand multiple cycles of invadopodia elongation and retraction during cancer invasion. Second, we then aim to understand the actin-myosin machinery in the IP dynamics by computationally knocking-out crosslinking proteins, such as α-actinin, filamin, and fascin.

To our knowledge, this is the first report on a 3D cancer invasive model for the prediction of either protrusion or inhibition of invadopodia by incorporating two-way interactions between the discrete intracellular branched actin network and ECM fiber network as well as knock-out simulations for actin-crosslinking molecules.

## Results

### Cancer cell invasion model with invadopodia

**Figure 1:**
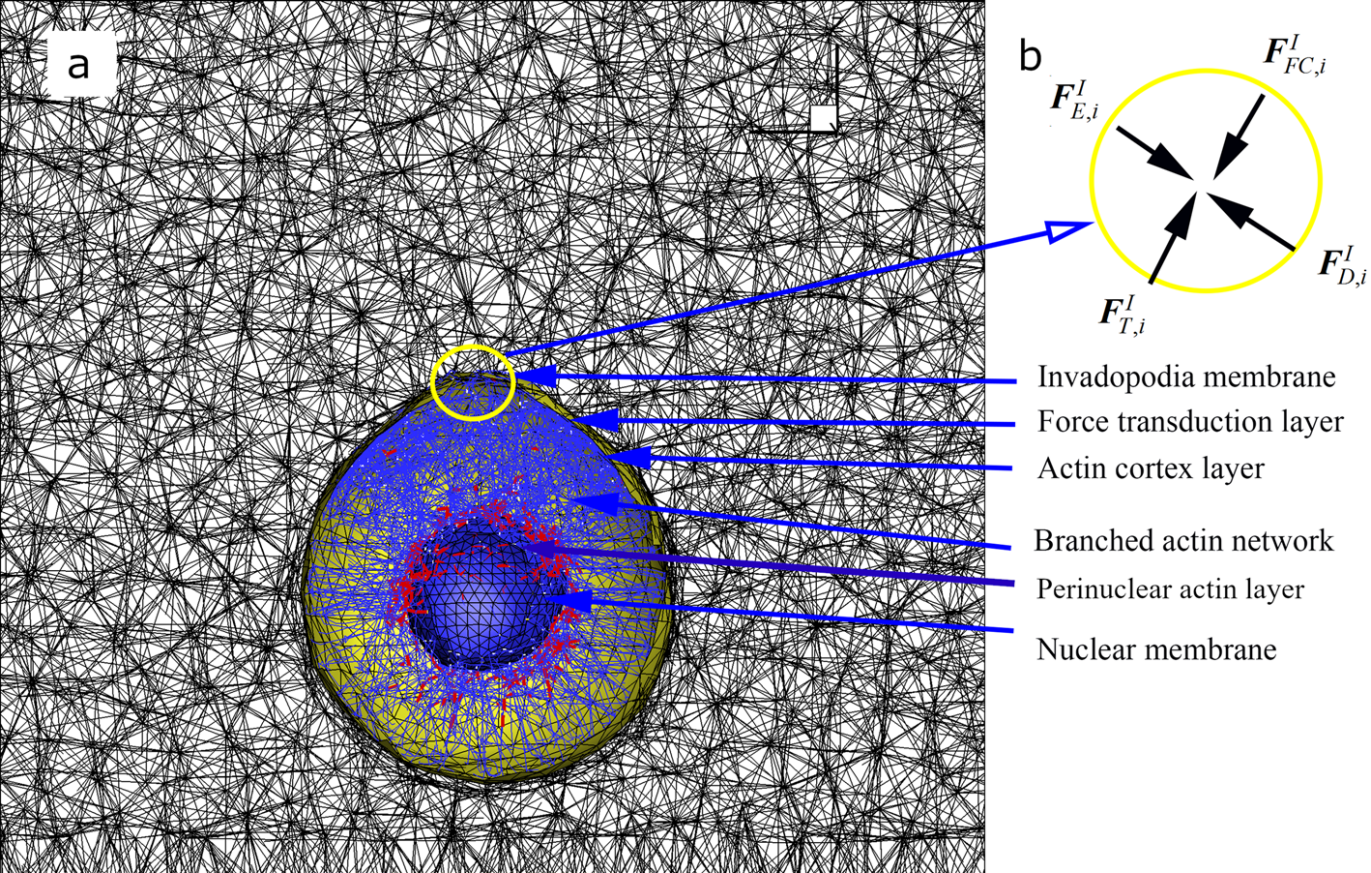
Dynamic model of cancer invadopodia protrusion in a viscoelastic ECM fiber network. (**a**) Integrated cancer cell migration model consisting of invadopodia membrane (yellow), force transduction layer (green), actin cortex layer, branched actin network, perinuclear actin layer, and nuclear membrane (blue). Viscoelastic behaviors in all of them are modeled using Kelvin-Voigt model. (**b**) The free body diagram of the *i*-th invadopodial node in the circle marked in (**a**) where four external forces are acting.

The activity of invadopodia, as well as its interaction with 3D ECM^13^, is a complicated, multifaceted process where at least three individual dynamics are coupled: (1) cell mechanics, including the protrusion and retraction of invadopodia membrane and the growth of discrete intracellular branched actin network; (2) mechanics of the discrete ECM fiber network (S1 Text); and (3) reaction–diffusion mass transfer over ECM (Supplementary Fig. 1 and Supplementary Information). In addition, to incorporate viscoelastic behaviors in (1) triple layers of cellular membrane (invadopodia membrane, force transduction layer (FTL), and actin cortex layer (ACL)), (2) intracellular branched actin network, and (3) double layers of nuclear membrane (perinuclear actin layer (PAL), and nuclear membrane surface (NMS)) (Fig. 1a), all the line elements in their structures were simulated with Kelvin-Voight model (a spring and a dashpot in parallel). Figure 1b shows the free body diagram of the *i*-th node on the invadopodia membrane, called the *i*-th integrin node, where a bundle of integrins is formed. The detailed equations that govern each of these dynamical processes and a list of simulation parameters are summarized in Table 1 and Supplementary Table 1, respectively.

**Table 1:** Dynamical model of invadopodia protrusion.

Module	Equation	Couplings
CI	$\underbrace {{\mathop \sum \limits_{j = 1}^{n\left( i \right)} C_{i,j}^{I} \left( {\frac{{d{\varvec{x}}_{i}^{I} }}{dt} - \frac{{d{\varvec{x}}_{j}^{I} }}{dt}} \right) + C_{1}^{T} \left( {\frac{{d{\varvec{x}}_{i}^{I} }}{dt} - \frac{{d{\varvec{x}}_{i}^{T} }}{dt}} \right) + C_{0}^{I} \frac{{d{\varvec{x}}_{i}^{I} }}{dt} = {\varvec{F}}_{E,i}^{I} + {\varvec{F}}_{FC,i}^{I} + {\varvec{F}}_{T,i}^{I} , i = 1, \cdots ,N_{I} }}_{{ - {\varvec{F}}_{D,i}^{I} }}$	E, CT, $\phi_{2}$,${ }\phi_{3}$ and ${ }\phi_{4}$
CT	$\underbrace {{\mathop \sum \limits_{j = 1}^{n\left( i \right)} C_{i,j}^{T} \left( {\frac{{d{\varvec{x}}_{i}^{T} }}{dt} - \frac{{d{\varvec{x}}_{j}^{T} }}{dt}} \right) + C_{1}^{T} \left( {\frac{{d{\varvec{x}}_{i}^{T} }}{dt} - \frac{{d{\varvec{x}}_{i}^{I} }}{dt}} \right) + C_{1}^{C} \left( {\frac{{d{\varvec{x}}_{i}^{T} }}{dt} - \frac{{d{\varvec{x}}_{i}^{C} }}{dt}} \right) + C_{0}^{T} \frac{{d{\varvec{x}}_{i}^{I} }}{dt} = {\varvec{F}}_{E,i}^{T} + {\varvec{F}}_{T,i}^{T} + {\varvec{F}}_{C,i}^{T} , i = 1, \cdots ,N_{T} }}_{{ - {\varvec{F}}_{D,i}^{T} }}$	CI and CC
CC	$\underbrace {{\mathop \sum \limits_{j = 1}^{n\left( i \right)} C_{i,j}^{C} \left( {\frac{{d{\varvec{x}}_{i}^{C} }}{dt} - \frac{{d{\varvec{x}}_{j}^{C} }}{dt}} \right) + C_{1}^{C} \left( {\frac{{d{\varvec{x}}_{i}^{C} }}{dt} - \frac{{d{\varvec{x}}_{i}^{T} }}{dt}} \right) + C_{0}^{C} \frac{{d{\varvec{x}}_{i}^{C} }}{dt} = {\varvec{F}}_{E,i}^{C} + {\varvec{F}}_{C,i}^{C} + {\varvec{F}}_{L,i}^{C} + {\varvec{F}}_{P,i}^{C} , i = 1, \cdots ,N_{C} }}_{{ - {\varvec{F}}_{D,i}^{C} }}$	CT and CA
CA	$\underbrace {{\mathop \sum \limits_{k = j - 1}^{j + 1} C_{i,j}^{A} \left( {\frac{{d{\varvec{x}}_{i}^{A} }}{dt} - \frac{{d{\varvec{x}}_{k}^{A} }}{dt}} \right) + C_{i,ARP}^{A} \left( {\frac{{d{\varvec{x}}_{i,j}^{A} }}{dt} - \frac{{d{\varvec{x}}_{k,1}^{A} }}{dt}} \right) + C_{0}^{A} \frac{{d{\varvec{x}}_{i,j}^{A} }}{dt} = {\varvec{F}}_{E,i,j}^{A} + {\varvec{F}}_{br,i,j}^{A} + {\varvec{F}}_{L,i,j}^{A} + {\varvec{F}}_{C,i,j}^{A} , i = 1, \cdots ,N_{A} }}_{{ - {\varvec{F}}_{D,i,j}^{A} }}$	CC and CN
CN	$\underbrace {{\mathop \sum \limits_{j = 1}^{n\left( i \right)} C_{i,j}^{N} \left( {\frac{{d{\varvec{x}}_{i}^{N} }}{dt} - \frac{{d{\varvec{x}}_{j}^{N} }}{dt}} \right) + C_{0}^{N} \frac{{d{\varvec{x}}_{i}^{N} }}{dt} = {\varvec{F}}_{E,i}^{N} + {\varvec{F}}_{L,i}^{N} , i = 1, \cdots ,N_{T} }}_{{ - {\varvec{F}}_{D,i}^{N} }}$	CA
E	$\left(2{C}_{ij}^{E}+{C}_{0}^{E}\right)\frac{\mathrm{d}{x}_{ij}^{E}}{\mathrm{dt}}={F}_{E,ij}^{E}$+${F}_{FC,ij}^{E}+{F}_{D,ij}^{E0}, i=1,\cdots ,{N}_{fiber}^{E}$	CI and ${\phi }_{6}$
RD		
${\phi }_{1}$	$\frac{\partial {\phi }_{1}}{\partial \mathrm{t}}=\nabla \cdot \left({D}_{{\phi }_{1}}\nabla {\phi }_{1}\right)-{k}_{{\phi }_{1}:{\phi }_{2}}^{on}{\phi }_{1}{\phi }_{2}+{k}_{{\phi }_{3}:{\phi }_{4}}^{on}{\phi }_{3}{\phi }_{4}-{k}_{{\phi }_{1}}^{decay}{\phi }_{1}$	E, ${\phi }_{2}$, ${\phi }_{3}$ and ${\phi }_{4}$
${\phi }_{2}$	$\frac{\partial {\phi }_{2}}{\partial \mathrm{t}}=\nabla \cdot \left({D}_{{\phi }_{2}}\nabla {\phi }_{2}\right)-{k}_{{\phi }_{1}:{\phi }_{2}}^{on}{\phi }_{1}{\phi }_{2}-{k}_{{\phi }_{2}:{\phi }_{3}}^{on}{\phi }_{2}{\phi }_{3}+{k}_{{\phi }_{4}}^{off}{\phi }_{4}+{\alpha }_{{\phi }_{2}}\left({x}_{tip}^{I}\right){\phi }_{5}$	E,CI,${\phi }_{1}$,${\phi }_{3}$,${\phi }_{4}$ and ${\phi }_{5}$
${\phi }_{3}$	$\frac{\partial {\phi }_{3}}{\partial \mathrm{t}}=-{k}_{{\phi }_{2}:{\phi }_{3}}^{on}{\phi }_{2}{\phi }_{3}+{k}_{{\phi }_{4}}^{off}{\phi }_{4}-{k}_{{\phi }_{3}}^{decay}{\phi }_{3}+{\alpha }_{{\phi }_{3}}\left({x}_{tip}^{I}\right){\phi }_{5}$	CI, ${\phi }_{2}$, ${\phi }_{4}$ and ${\phi }_{5}$
${\phi }_{4}$	$\frac{\partial {\phi }_{4}}{\partial \mathrm{t}}={k}_{{\phi }_{2}:{\phi }_{3}}^{on}{\phi }_{2}{\phi }_{3}+{k}_{{\phi }_{3}:{\phi }_{4}}^{on}{\phi }_{3}{\phi }_{4}-{k}_{{\phi }_{4}}^{off}{\phi }_{4}$	CI, ${\phi }_{2}$ and ${\phi }_{3}$
${\phi }_{5}$	$\frac{\partial {\phi }_{5}}{\partial \mathrm{t}}=\nabla \cdot \left({D}_{{\phi }_{5}}\nabla {\phi }_{5}\right)-{k}_{{\phi }_{5}}^{decay}{\phi }_{5}+{k}_{{\phi }_{6}}^{deg}{\phi }_{1}{\phi }_{6}$	${\phi }_{1}$ and ${\phi }_{6}$
${\phi }_{6}$	$\frac{\partial {\phi }_{6}}{\partial \mathrm{t}}=-{k}_{{\phi }_{6}}^{deg}{\phi }_{1}{\phi }_{6}$	E and ${\phi }_{1}$

*MMP-2 (${\phi }_{1}$), TIMP-2 (${\phi }_{2}$), MT1-MMP (${\phi }_{3}$), a ternary complex of MT1-MMP:TIMP-2:proMMP-2 (${\phi }_{4}$), ligands (${\phi }_{5}$) (or collagen molecules) and ECM (${\phi }_{6}$). The three modules describe the dynamics of the cell (C) and ECM (E) modules and the reaction–diffusion (RD) modules. C module: C is composed of five sub-modules representing the invadopodia membrane (CI), the force transduces layer (CT), the actin cortex layer (CC), branched actin network (CA) and the double layers of nuclear membrane dynamics (CN). Detailed explanation of Table 1 can be found in Supplementary Information.

### Invadopodia dynamics

**Figure 2:**
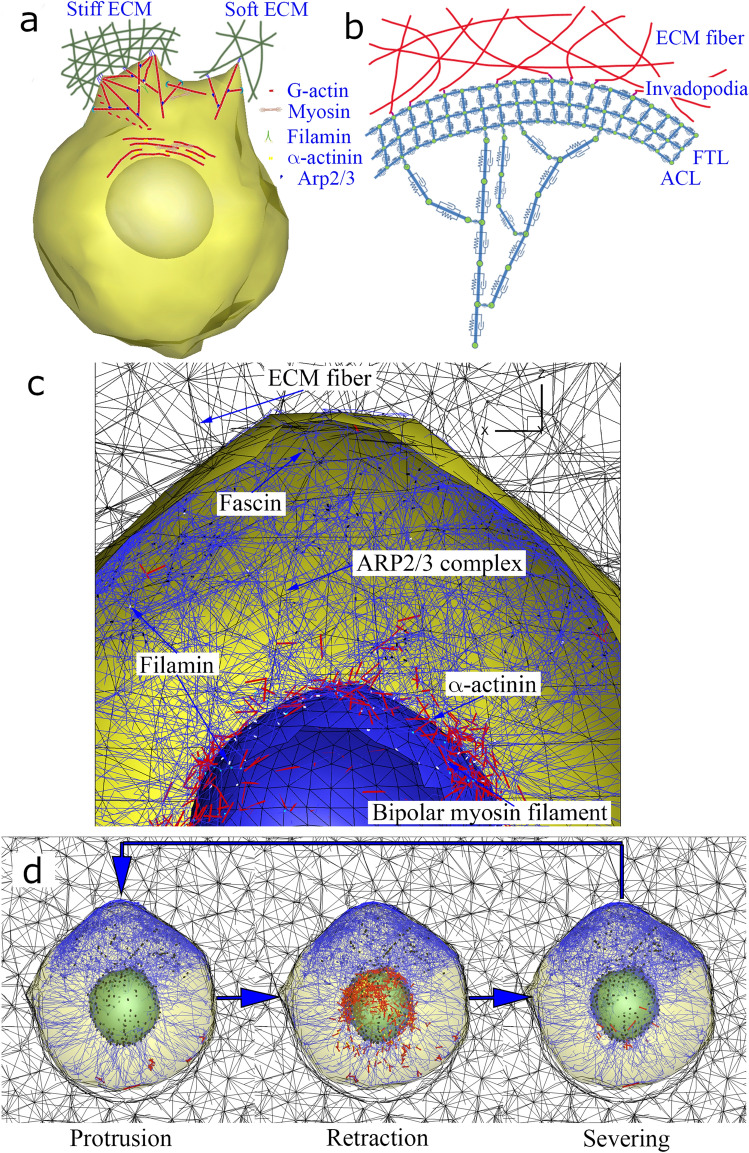
Computational model of invadopodia protrusion. (**a**) Schematic of invadopodia protrusions into stiff and soft ECMs showing the adaptation of branched actin network by surrounding ECM stiffness or density. (**b**) A schematic showing viscoelastic behaviors in the branched actin network, actin cortex layer (ACL), force transduction layer (FTL), cellular membrane, and ECM fiber network, which are modeled using Kelvin-Voight model (a spring and a dashpot together in parallel). (**c**) Schematic shows mechanical interactions between intracellular branched actin filaments and ECM fiber network via invadopodia protrusion. Three kinds of actin-crosslinkers, such as filamin, α-actinin, and fascin, connect two neighboring actin filaments. Additionally, actin filament and perinuclear actin layer (PAL) on the nuclear membrane surface (NMS) can be also connected by filamin and α-actinin. ARP2/3 complex nucleates a new branched actin filament (daughter) from the existing filament (mother), and bipolar myosin filaments slide on two neighboring actin filaments to gain contractile forces. (**d**) Invadopodia protrusion dynamics showing protrusive, retractile, and severing phases.

Dynamic behaviors of invadopodia and their interactions with the network of intracellular F-actins are complex, but can be predicted with a computational model based on a few assumptions and observations. First, the discrete branched actin network is composed of viscoelastic actin filaments (diameter of 7 nm and modulus of 1.8 GPa)^14^, Arp2/3 complexes, capping proteins (CP), actin crosslinking molecules (α-actinin, filamin, and fascin), and contractile bipolar myosin filaments (Fig. 2a–c). Second, the invadopodia protrusion dynamics into 3D ECM consist of three different motion phases in a single cycle (Fig. 2d)^12^: 1) a protrusive phase driven by F-actin polymerization, 2) a retractile phase due to contractile motions of perinuclear bipolar myosin filaments, and (3) a severing phase^15^ for the rapid depolymerization of buckled actin filaments^16^ by actin severing proteins (Cofilin and Gelsolin). Particularly, the duration of protrusive phase has a key role in facilitating invadopodia protrusion into ECM since MT1-MMP, which is accumulated at tips of invadopodia, can degrade surrounding ECM only during this phase. Accordingly, MT1-MMP signal pathway is assumed to be switched on only during this phase.

### Protrusive phase in discrete F-actin mechanics

Here, we model invadopodia protrusion induced by the growth of branched actin network. To incorporate the polymerization at the barbed-end for *i*-th actin filament (${{\varvec{x}}}_{i,{n}_{i}^{A}}^{A}$ where ${n}_{i}^{A}$ indicates number of nodes in *i*-th actin filament) and the depolymerization at the pointed-end of *i*-th actin filament (${\varvec{x}}_{i,1}^{A}$), it is assumed that an unstressed length of the *j*-th line element of the *i*-th actin filament,$L_{i,j}^{A0}$, is updated every time-step of the simulation with two rates of $L_{i,j}^{A0}$ with time; one is $\frac{d{L}_{i,{n}_{i}^{A}-1}^{A0}}{dt}={v}_{g}$ at the barbed-end for actin filament, and the other is $\frac{d{L}_{i,1}^{A0}}{dt}={-v}_{s}$ at the pointed-end of actin filament where $v_{g}$ is growing rate and $v_{s}$ is shrinkage rate of 10 nm/s^17^. $v_{g}$ is constant or variable depending on the adhesion of barbed-end for actin filament to the actin cortex layer (ACL). $v_{g}$ is constant (20 nm/s) when a daughter actin filament is nucleated from a mother filament. Then, as growing actin filament starts interacting with the surface of ACL, $v_{g}$ becomes variable. To model the mechanics of growing actin filament against the surface of ACL, concave-up velocity-force relation (exponential decrease of the velocity with the growing load) is used^18^. Here ${v}_{g}={v}_{0}exp\left(-\frac{{f}_{cortex}}{{{n}_{F} f}_{0}}\right)$ where ${v}_{0}$ is 50 nm/s, ${f}_{cortex}$ is the tension at a node on the ACL, ${n}_{F}$ is the number of actin filaments interacting with the node on the ACL, and ${f}_{0}$ is 20 pN. Thereby, the effective stiffness of the *j*-th line elements of the *i*-th actin filament is variable,$\kappa_{L,i,j}^{A} = \frac{{A_{A} E_{A} }}{{L_{i,j}^{A0} }},$ where $E_{A}$ is the Young’s modulus of actin filament (1.8 GPa)^14^ and $A_{A}$ is the average cross-sectional area of actin filament (38.48 ${\mathrm{nm}}^{2}$). It should be noted that an initial value of $L_{i,j}^{A0}$ is 150 nm, and thus an initial value of ${\kappa }_{L,i,j}^{A}$ is 0.46 N/m.

**Figure 3:**
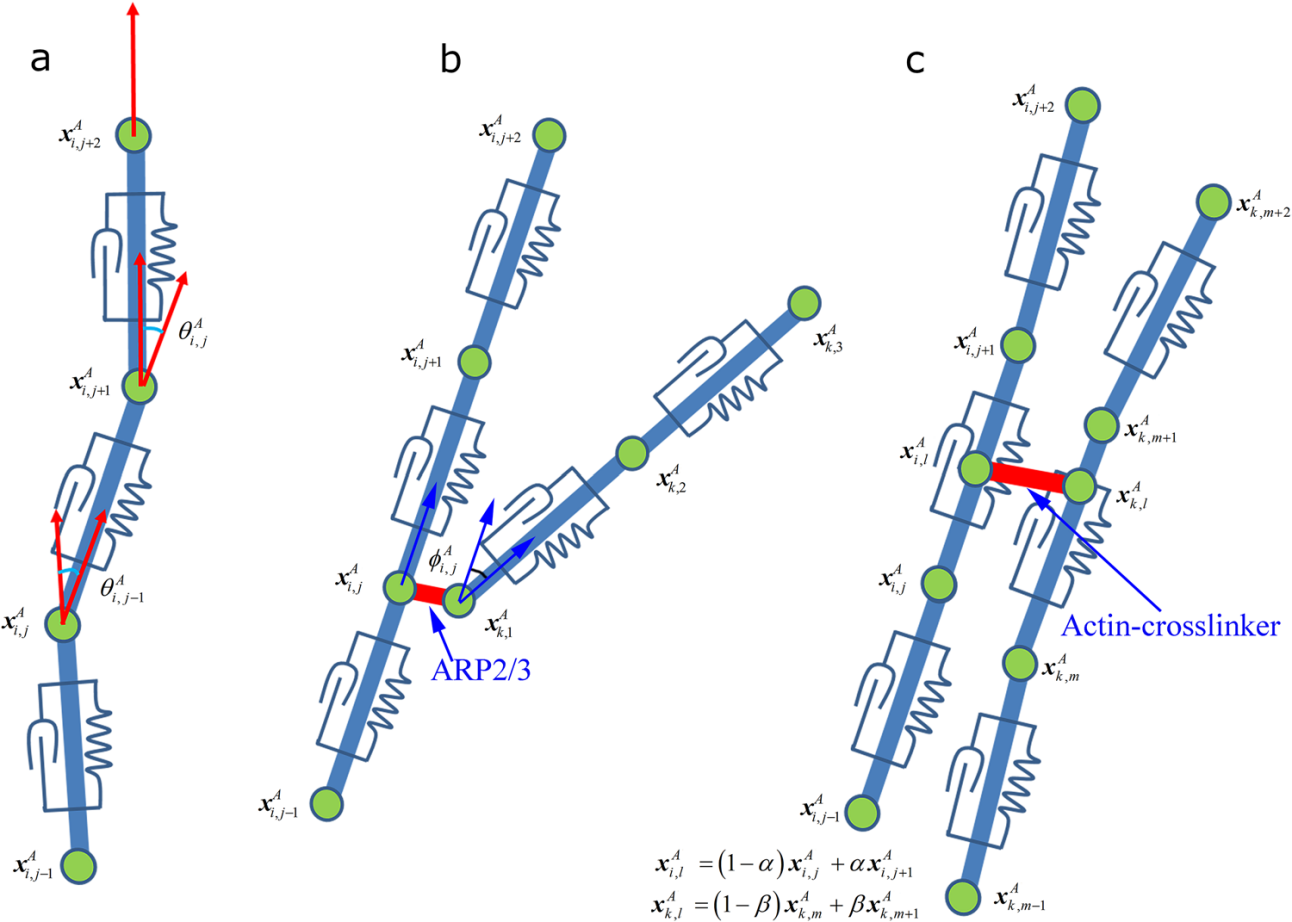
Mechanical interactions in viscoelastic actin filaments. (**a**) A schematic representation of single actin filament with series of springs and dashpots for the calculation of two kinds of elastic forces due to stretching and bending actin filament. (**b**) A schematic representation of branched actin filament for the calculation of two kinds of elastic forces due to branched and dihedral angles. ${\varvec{x}}_{i\,,j}^{AF}$ and ${\varvec{x}}_{k\,,1}^{AF}$ indicate binding sites of ARP2/3 complex. (**c**) A schematic representation of cross-linked actin filaments. ${\varvec{x}}_{i\,,l}^{AF}$ and ${\varvec{x}}_{k\,,l}^{AF}$ indicate binding sites of actin-crosslinker.

In our previous publication, we applied the virtual energy method in structure mechanics to derive elastic forces at the cellular and nuclear membranes^19,20^, and ECM fibers^21,22^. Here, in a similar manner, we apply the virtual energy method to derive elastic forces at actin network include F-actin, crosslinking molecules, and myosin minifilaments. In addition, a previous study used MEDYAN model and energy potential method to calculate elastic forces due to stretching and bending polymer as well as branching and dihedral angles^23^. It should be noted that our method is different from theirs in that we analytically derive elastic force by differentiating total elastic energy at the specified node. Here, the total elastic energy stored in the *i*-th actin filament is given by

1 $${H}_{L,i}^{A}=\frac{1}{2}\sum_{j=1}^{{N}_{i,{n}_{i}^{A}}^{A}-1}{\kappa }_{L,i,j}^{A}{\left({L}_{i,j}^{A}-{L}_{i,j}^{A0}\right)}^{2}$$

where $L_{i,j}^{A}$ is the length of the *j*-th line of the *i*-th actin filament and is updated at every time-step ($L_{i,j}^{A} = \left\| {{\varvec{x}}_{i,j + 1}^{A} - {\varvec{x}}_{i,j}^{A} } \right\|$, where ${{\varvec{x}}}_{i,j}^{A}$ and ${{\varvec{x}}}_{i,j+1}^{A}$ are location vectors of the *j*-th and *j* + 1-th nodes of the *i*-th actin filament) (Fig. 3a). $L_{i,j}^{A0}$ is its relaxed length (150 nm). The other is the total elastic energy associated with the change of angle between $L_{i,j}^{A}$ and $L_{i,j + 1}^{A}$ on the *i*-th actin filament, and it is expressed as

2 $${H}_{B,i}^{A}=\frac{1}{2}\sum_{j=1}^{{N}_{i,{n}_{i}^{A}}^{A}-2}{\kappa }_{b}^{A}\frac{{\left({\uptheta }_{i,j}^{A}-{\uptheta }_{i,j}^{A0}\right)}^{2}}{{L}_{i,j}^{A0}}$$

where $\kappa_{b}^{A}$ is the bending modulus of actin filament is expressed as $\kappa_{b}^{A} = E_{A}^{{}} I_{A}$, where $I_{A} \left( { = {{\pi r_{A}^{4} } \mathord{\left/ {\vphantom {{\pi r_{A}^{4} } 4}} \right. \kern-\nulldelimiterspace} 4}} \right)$, $\theta_{i\,,j}^{A}$ and $\theta_{i\,,j}^{A0}$ are the stressed and unstressed angles (zero) between $L_{i,j}^{A}$ and $L_{i,j + 1}^{A}$ on the *i*-th actin filament, respectively (Fig. 3a). Similarly, the elastic force at the *j*-th node on the *i*-th actin filament, ${{\varvec{F}}}_{E,i,j}^{A}$, can be derived by using the virtual work theory:

3 $${{\varvec{F}}}_{E,i,j}^{A}=-\frac{\partial {H}_{L,i}^{A}}{\partial {{\varvec{x}}}_{i,j}^{A}}-\frac{\partial {H}_{B,i}^{A}}{\partial {{\varvec{x}}}_{i,j}^{A}}=-\sum_{k=j}^{j+1}{\kappa }_{L,i,k}^{A}\frac{\left({L}_{i,k}^{A}-{L}_{i,k}^{A0}\right)}{{L}_{i,k}^{A0}}\frac{\partial {L}_{i,k}^{A}}{\partial {{\varvec{x}}}_{i,j}^{A}}-\sum_{k=j-1}^{j+1}{\kappa }_{b}^{A}\frac{\left({\uptheta }_{i,k}^{A}-{\uptheta }_{i,k}^{A0}\right)}{{L}_{i,k}^{A0}}\frac{\partial {\uptheta }_{i,k}^{A}}{\partial {{\varvec{x}}}_{i,j}^{A}}$$

where $\theta_{i,k}^{A} = \cos^{ - 1} \left( {\hat{\user2{t}}_{i,k} \cdot \hat{\user2{t}}_{i,k + 1} } \right),$ $\hat{\user2{t}}_{i,k}$ and $\hat{\user2{t}}_{i,k + 1}$ are tangential unit vectors at the *k* and *k* + 1-th lines in the *i*-th actin filament, respectively, and

$$ \frac{{\partial \theta_{i\,j}^{A} }}{{\partial {\varvec{x}}_{i\,,j}^{A} }} = \frac{ - 1}{{\sqrt {1 - \left( {\hat{\user2{t}}_{i,j} \cdot \hat{\user2{t}}_{i,j + 1} } \right)^{2} } }}\left( {\frac{{\partial \hat{\user2{t}}_{i,j} }}{{\partial {\varvec{x}}_{i,\,j}^{A} }} \cdot \hat{\user2{t}}_{i,j + 1} + \hat{\user2{t}}_{i,j} \cdot \frac{{\partial \hat{\user2{t}}_{i,j + 1} }}{{\partial {\varvec{x}}_{i\,j}^{A} }}} \right). $$

In case that a new *k*-th actin filament (daughter) is nucleated from the Arp2/3 complex which is on the *j*-th node on the *i*-th actin filament (mother), an elastic Arp2/3 force,${\varvec{F}}_{arp,i,j}^{A}$, and elastic branch force,${\varvec{F}}_{br,i,j}^{A}$, are non-zero (Fig. 3b). Similarly,${\varvec{F}}_{arp,i,j}^{A}$ is expressed as

4 $${{\varvec{F}}}_{arp,i,j}^{A}=-{\kappa }_{arp}\left({L}_{arp,i,j}^{A}-{\lambda }_{arp}\right)\frac{\partial {L}_{arp,i,j}^{A}}{\partial {{\varvec{x}}}_{i,j}^{A}}$$

where $\kappa_{arp}$ is the effective stiffness constants of Arp2/3 complex (0.01 N/m), $L_{arp,i,j}^{A}$ is the distance between the *j*-th node on the *i*-th actin filament and the first node on the *k*-th actin filament, and $\lambda_{arp}$ is unstressed length of Arp2/3 complex (30 nm). ${\varvec{F}}_{br,i,j}^{A}$ has two kinds of branch forces: one is branch angle force, and the other is branch dihedral force^23^. Total branch elastic energy at the *j*-th node on the *i*-th actin filament is expressed as

5 $$ {\rm H}_{BR,i,j}^{A} = \frac{1}{2}\kappa_{br,ang}^{A} \frac{{\left( {\phi_{i,\,j}^{A} - \phi_{i\,,j}^{A0} } \right)^{2} }}{{L_{arp,i,j}^{A0} }} + \frac{1}{2}\kappa_{br,dihed}^{A} \frac{{\left( {\psi_{i\,,j}^{A} - \psi_{i\,,j}^{A0} } \right)^{2} }}{{L_{arp,i,j}^{A0} }} $$

where $\kappa_{br,ang}^{A}$ is the angular bending modulus^23^, $\phi_{i,\,j}^{A}$ is the branched angle between $L_{i,j}^{A}$ and $L_{k,1}^{A}$. That is, the angle between two unit vectors of $\hat{\user2{l}}_{i,j} \left( { = \frac{{{\varvec{x}}_{i,j + 1}^{A} - {\varvec{x}}_{i,j}^{A} }}{{\left\| {{\varvec{x}}_{i,j + 1}^{A} - {\varvec{x}}_{i,j}^{A} } \right\|}}} \right)$ and $\hat{\user2{l}}_{k,1} \left( { = \frac{{{\varvec{x}}_{k,2}^{A} - {\varvec{x}}_{k,1}^{A} }}{{\left\| {{\varvec{x}}_{k,2}^{A} - {\varvec{x}}_{k,1}^{A} } \right\|}}} \right)$, and $\phi_{i\,,j}^{A0}$ is an equilibrium branched angle ~ 70˚ between the mother and daughter filaments. $\kappa_{br,dihed}^{A}$ is the dihedral bending modulus^23^, $\psi_{i,\,j}^{A}$ is the dihedral angle between two planes, formed by the points $\left( {{\varvec{x}}_{i,j + 1}^{A} ,{\varvec{x}}_{i,j}^{A} ,{\varvec{x}}_{k,1}^{A} } \right)$ and $\left( {{\varvec{x}}_{i,j}^{A} ,{\varvec{x}}_{k,1}^{A} ,{\varvec{x}}_{k,2}^{A} } \right)$, and $\psi_{i\,,j}^{A0}$ is assumed to be zero. Two vectors normal to the planes are respectively expressed as $\hat{\user2{m}}_{i,j} = \frac{{{\varvec{x}}_{i,j}^{A} - {\varvec{x}}_{k,1}^{A} }}{{L_{ARP,i,j}^{A} }} \times \hat{\user2{l}}_{i,j} ,\,\hat{\user2{m}}_{k,1} = \frac{{{\varvec{x}}_{i,j}^{A} - {\varvec{x}}_{k,1}^{A} }}{{L_{ARP,i,j}^{A} }} \times \hat{\user2{l}}_{k,1}$. Thereby, ${\varvec{F}}_{br,i,j}^{A}$ can be derived by using the virtual work theory:

6 $$ {\varvec{F}}_{br,i\,,j}^{A} = - \kappa_{br,ang}^{A} \frac{{\left( {\phi_{i\,,j}^{A} - \phi_{i,\,j}^{A0} } \right)}}{{L_{ARP,i,j}^{A0} }}\frac{{\partial \phi_{i,\,j}^{A} }}{{\partial {\varvec{x}}_{i\,,j}^{A} }} - \kappa_{br,dihed}^{A} \frac{{\left( {\psi_{i\,,j}^{A} - \psi_{i\,,j}^{A0} } \right)}}{{L_{ARP,i,j}^{A0} }}\frac{{\partial \psi_{i\,,j}^{A} }}{{\partial {\varvec{x}}_{i,\,j}^{A} }} $$

where $\phi_{i,\,j}^{A} = \cos^{ - 1} \left( {\hat{\user2{l}}_{i,j} \cdot \hat{\user2{l}}_{k,1} } \right),$ and $\psi_{i,\,j}^{A} = \cos^{ - 1} \left( {\hat{\user2{m}}_{i,j} \cdot \hat{\user2{m}}_{k,1} } \right)$. Similarly, $\frac{{\partial \phi_{i,\,j}^{A} }}{{\partial {\varvec{x}}_{i\,,j}^{A} }}$ and $\frac{{\partial \psi_{i\,,j}^{A} }}{{\partial {\varvec{x}}_{i,\,j}^{A} }}$ are respectively expressed as $\frac{{\partial \phi_{i,\,j}^{A} }}{{\partial {\varvec{x}}_{i\,,j}^{A} }} = \frac{ - 1}{{\sqrt {1 - \left( {\hat{\user2{l}}_{i,j} \cdot \hat{\user2{l}}_{k,1} } \right)^{2} } }}\left( {\frac{{\partial \hat{\user2{l}}_{i,j} }}{{\partial {\varvec{x}}_{i,\,j}^{A} }} \cdot \hat{\user2{l}}_{k,1} + \hat{\user2{t}}_{i,j} \cdot \frac{{\partial \hat{\user2{l}}_{k,1} }}{{\partial {\varvec{x}}_{i\,,j}^{A} }}} \right)$ and $\frac{{\partial \psi_{i\,,j}^{A} }}{{\partial {\varvec{x}}_{i,\,j}^{A} }} = \frac{ - 1}{{\sqrt {1 - \left( {\hat{\user2{m}}_{i,j} \cdot \hat{\user2{m}}_{k,1} } \right)^{2} } }}\left( {\frac{{\partial \hat{\user2{m}}_{i,j} }}{{\partial {\varvec{x}}_{i,\,j}^{A} }} \cdot \hat{\user2{m}}_{k,1} + \hat{\user2{m}}_{i,j} \cdot \frac{{\partial \hat{\user2{m}}_{k,1} }}{{\partial {\varvec{x}}_{i\,,j}^{A} }}} \right).$

Furthermore, a polymerization force due to Brownian ratchet motion^18,24^, which is induced by thermal fluctuations of membrane, is also incorporated in the model, and it is expressed as

7 $${{\varvec{F}}}_{P,i}^{C}={F}_{p}log\left(\frac{{v}_{0}}{\Vert {\widehat{{\varvec{n}}}}_{R,i}^{I}\cdot  \left({{\varvec{v}}}_{b}-{{\varvec{v}}}_{a}\right)\Vert }\right)$$

where $F_{p}$ is assumed to be 20 pN because the force exerted by each actin filament is the order of 10 ~ 20 pN^25^, $v_{0}$ is zero load polymerization speed of actin filament (50 nm/s), $\hat{\user2{n}}_{R,i}^{I}$ is a unit vector normal to the surface of *i*-th invadopodial membrane, ${\varvec{v}}_{b}$ is a velocity vector at the barbed-end for actin filament (CA module), and ${\varvec{v}}_{a}$ is a velocity vector at the node of actin cortex layer mesh (CC module) where barbed-end for actin filament interacted with. It should be noted that this polymerization process due to Brownian ratchet motion is switched on during the protrusive phase, but it is switched off during both the retractile and severing phases since binding of capping proteins (CPs) at barbed-ends for actin filaments block the addition or loss of G-actins.

### Retractile phase in discrete F-actin mechanics

To incorporate the retractile phase in the invadopodia dynamics, mechanical interactions of 3D branched actin filaments by actin crosslinking molecules (fascin, filamin and α-actinin) and contractile bipolar myosin filaments are modelled. We assume that fascins connect two parallel adjacent actin filaments which are bundled in the core of invadopodia, and filamins connect two parallel or orthogonal actin filaments which are located in the leading edge of the cell. It should be noted that both fascins and filamins were assumed to form in the leading edge of the cell, where their directional angles between a unit vector of cellular polarity (migration direction) and their location vectors from the center of cell were less than 60 degrees. In case of α-actinin and bipolar myosin filaments, they are assumed to be activated during the retractile phase and connect two parallel actin filaments which surround the nucleus^26^. The elastic energy at the *l*-th actin-crosslinker, which connect the *j*-th line on the *i*-th actin filament and the *m*-th line on the *k*-th actin filament (Fig. 3c), is expressed as

8 $$ H_{L,l}^{A} = \frac{1}{2}\kappa_{L}^{A} \left( {L_{l}^{A} - L_{l}^{A0} } \right)^{2} $$

where $\kappa_{L}^{A}$ is the effective stiffness constant of the actin-crosslinkers (0.01 N/m), and $L_{l}^{A} = \left\| {{\varvec{x}}_{i\,,l}^{A} - {\varvec{x}}_{k\,,l}^{A} } \right\|$ and $L_{l}^{0}$ are the stressed and unstressed lengths of the *l*-th actin-crosslinker, respectively. Here ${\varvec{x}}_{i\,,l}^{A}$ and ${\varvec{x}}_{k\,,l}^{A}$ indicate binding positions on the *j*-th line on the *i*-th actin filament and the *m*-th line on the *k*-th actin filament, respectively. They are expressed as ${\varvec{x}}_{i\,,l}^{A} = \left( {1 - \alpha } \right){\varvec{x}}_{i\,,j}^{A} + \alpha {\varvec{x}}_{i\,,j + 1}^{A}$ and ${\varvec{x}}_{k\,,l}^{A} = \left( {1 - \beta } \right){\varvec{x}}_{k\,,m}^{A} + \beta {\varvec{x}}_{k\,,m + 1}^{A}$ ^23^. Here $\alpha \in \left[ {0,1} \right]$ and $\beta \in \left[ {0,1} \right]$ represent stochastic fractional ratios. Thereby, linking elastic forces at the *j*-th node on the *i*-th actin filament,${\varvec{F}}_{L,i,j}^{A}$, can be derived by using virtual energy method, and it is expressed as

9a $$ {\varvec{F}}_{L,i,j}^{A} = - \left( {1 - \alpha } \right)\kappa_{L}^{A} \left( {L_{l}^{A} - L_{l}^{A0} } \right)\frac{{\partial L_{l}^{A} }}{{\partial {\varvec{x}}_{i,l}^{A} }} $$

Similarly, the linking elastic force at the *m*-th node on the *k*-th actin filament, ${\varvec{F}}_{L,k,m}^{A}$, can be expressed as

9b $$ {\varvec{F}}_{L,k,m}^{A} = - \left( {1 - \beta } \right)\kappa_{L}^{A} \left( {L_{l}^{A} - L_{l}^{A0} } \right)\frac{{\partial L_{l}^{A} }}{{\partial {\varvec{x}}_{k,l}^{A} }} $$

It has been known that a bipolar myosin filament move towards barbed-ends for two adjacent actin filaments. The elastic energy at the *l*-th bipolar myosin filament, which connect the *j*-th line element on the *i*-th actin filament and the *m*-th line element on the *k*-th actin filament, is expressed as

10 $$ H_{C,l}^{BMF} = \frac{1}{2}\kappa_{C}^{BMF} \left( {L_{l}^{BMF} - L_{l}^{BMF0} } \right)^{2} $$

where $\kappa_{C}^{BMF}$ is the effective stiffness constant of the bipolar myosin filament (0.01 N/m), and $L_{l}^{BMF} = \left\| {{\varvec{x}}_{i\,,l}^{A} - {\varvec{x}}_{k\,,l}^{A} } \right\|$ and $L_{l}^{BMF0}$ are the stressed and unstressed lengths of the *l*-th bipolar myosin filament (300 nm)^27^, respectively. Similarly, ${\varvec{x}}_{i\,,l}^{A}$ and ${\varvec{x}}_{k\,,l}^{A}$ indicate binding positions on the *j*-th line element on the *i*-th actin filament and the *m*-th line element on the *k*-th actin filament, respectively. They are expressed as ${\varvec{x}}_{i\,,l}^{A} = \left( {1 - \alpha } \right){\varvec{x}}_{i\,,j}^{A} + \alpha {\varvec{x}}_{i\,,j + 1}^{A}$, and ${\varvec{x}}_{k\,,l}^{A} = \left( {1 - \beta } \right){\varvec{x}}_{k\,,m}^{A} + \beta {\varvec{x}}_{k\,,m + 1}^{A}$. Similarly, contractile force at the *j*-th node on the *i*-th actin filament by a bipolar myosin filament, ${\varvec{F}}_{C,i,j}^{A}$, can be expressed as

11a $$ {\varvec{F}}_{C,i,j}^{A} = - \left( {1 - \alpha } \right)\kappa_{C}^{BMF} \left( {L_{l}^{BMF} - L_{l}^{BMF0} } \right)\frac{{\partial L_{l}^{BMF} }}{{\partial {\varvec{x}}_{i,l}^{A} }} $$

Contractile force at the *m*-th node on the *k*-th actin filament by a bipolar myosin filament, ${\varvec{F}}_{C,k,m}^{A}$, can be expressed as

11b $$ {\varvec{F}}_{C,k,m}^{A} = - \left( {1 - \beta } \right)\kappa_{C}^{BMF} \left( {L_{l}^{BMF} - L_{l}^{BMF0} } \right)\frac{{\partial L_{l}^{BMF} }}{{\partial {\varvec{x}}_{i,l}^{A} }} $$

It should be noted that $\alpha$ and $\beta$ are updated at every time-step because of sliding motions along to two actin filaments. Let ${\varvec{x}}_{i\,,l}^{A*}$ be the predicted binding position at the next time, ${\varvec{x}}_{i\,,l}^{A}$ represents the biding position at the current time, and it can be expressed as

12 $$ {\varvec{x}}_{i\,,l}^{A*} = {\varvec{x}}_{i\,,l}^{A} + v_{sliding} \vartriangle t\,\,\hat{\user2{t}}_{i,j} $$

where $v_{sliding}$ is the sliding rate of bipolar myosin filament, and $\vartriangle t\,$ is the time-step. Let $\alpha^{*}$ be the predicted $\alpha$ at the next time-step, and it is expressed as

13a $$ \alpha^{*} = \frac{{\left\| {{\varvec{x}}_{i\,,l}^{A*} - {\varvec{x}}_{i\,,j}^{A} } \right\|}}{{\left\| {{\varvec{x}}_{i\,,j + 1}^{A} - {\varvec{x}}_{i\,,j}^{A} } \right\|}} = \frac{{\sqrt {\left( {\alpha \left( {{\varvec{x}}_{i\,,j + 1}^{A} - {\varvec{x}}_{i\,,j}^{A} } \right) + v_{sliding} \vartriangle t\,\,\hat{\user2{t}}_{i,j} } \right) \cdot \left( {\alpha \left( {{\varvec{x}}_{i\,,j + 1}^{A} - {\varvec{x}}_{i\,,j}^{A} } \right) + v_{sliding} \vartriangle t\,\,\hat{\user2{t}}_{i,j} } \right)} }}{{\left\| {{\varvec{x}}_{i\,,j + 1}^{A} - {\varvec{x}}_{i\,,j}^{A} } \right\|}} $$

The above equation can be further expanded and simplified as following:

13b $$ \alpha^{*} = \alpha + \frac{{v_{sliding} }}{{\left\| {{\varvec{x}}_{i\,,j + 1}^{A} - {\varvec{x}}_{i\,,j}^{A} } \right\|}}\vartriangle t $$

**Figure 4:**
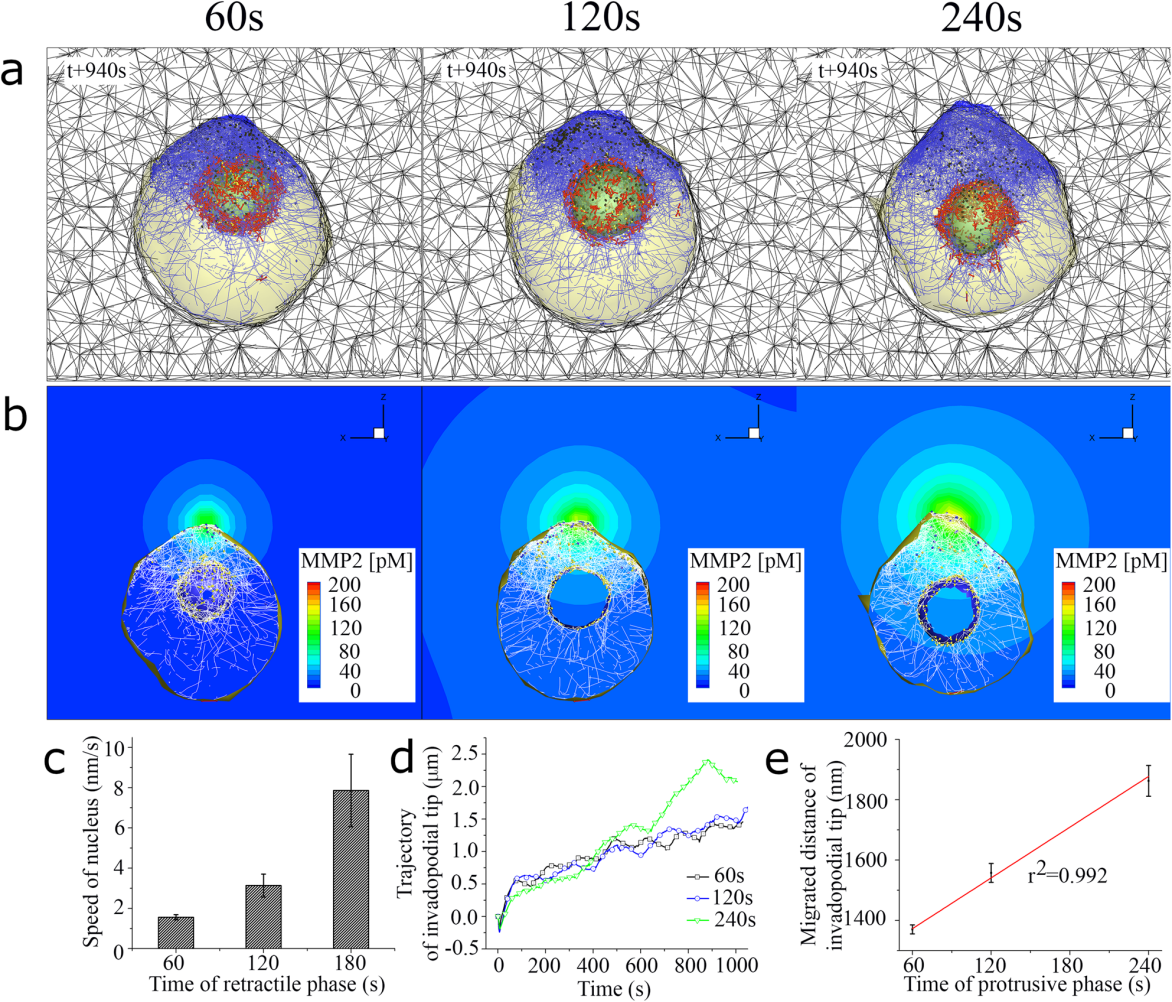
Characterization of invadopodia protrusion. (**a**) Selected still shots of simulated invadopodia protrusion into ECM under three different duration times in the protrusive phase, such as 60, 120, and 240 s. Blue, red, yellow, dark blue, and black lines indicate F-actin, bipolar myosin filament, α-actinin, filamin, and fascin, respectively. (**b**) Selected MMP-2 contour plots under three different duration times in the protrusive phase, such as 60, 120, and 240 s. (**c**) A graph showing speed of invadopodia verses duration time of retractile phase. (**d**) A graph showing z coordinate of invadopodia tip by time, and (**e**) linear regression (r^2^ = 0.992) between migrated distance of invadopodial tip and duration time of protrusive phase.

In addition, to incorporate mechanical interplay between the non-muscle myosin II and actin filaments, we adopt the force–velocity relation of muscle myosin II, first proposed by A.V. Hill^28^, and $v_{sliding}$ in Eq. (12) can be expressed as

14 $$ v_{sliding} = v_{sliding0} \frac{{F_{stall} - F_{ext} }}{{F_{stall} + c_{m} F_{ext} }} $$

where $v_{sliding0}$ is the sliding rate of myosin in the absence of load (160 nm/s), $F_{stall}$ is the stall force (1.24 Nn), $c_{m}$ is a dimensionless myosin parameter (4.761)^32^, and $F_{ext}$ is the magnitude of sensed contractile force at the previous time-step. The retractile phase is related with the disassembly of non-muscle myosin II with alpha-actinins. At the end of the retractile phase, α-actinins were assumed to turnover to terminate the contractile activity of non-muscle myosin II. Thereby, the time duration in the retractile phase means turnover time of α-actinins. In addition, we also modelled the turnover of fascin and filamin to incorporate dynamic behavior due to their rupturing and rebinding to two adjacent actin filaments. After parameter study on turnover time of α-actinin (see Fig. 4c), we assume to set turnover times of crosslinking proteins as 60 s, which is reasonable since measured lifetimes of the α-actinin/actin interactions range from 15 to 155 s. For example, by molecular rupture measurement under constant load, lifetime of the α-actinin/actin interaction was found to be ~ 20 s (corresponding to ${k}_{off}^{actinin}\approx 0.05 {\mathrm{s}}^{-1}$)^29^. Interestingly, intrinsic dissociation rates for α-actinin and filamin, which were estimated with theoretical model using overall rupture-force probability distributions, are 0.066 ± 0.028 ${\mathrm{s}}^{-1}$ and 0.087 ± 0.073 ${\mathrm{s}}^{-1}$, respectively^30^. Furthermore, lifetime of the disease-causing mutant K255E of human α-actinin-4 was measured as (155.1 s)^31^.

### Severing phase in discrete F-actin mechanics

Severing phase has a key role in the rapid depolymerization of buckled actin filaments^16^ resulting from contractile motions of perinuclear bipolar myosin filaments. As actin severing proteins (Cofilin and Gelsolin) can enhance disassembly of single actin filament by creating multiple pointed-ends of segmented actin filaments, a parallel mode of depolymerization process occurs at these pointed-ends of multiple actin filaments simultaneously. Thereby, severing phase with the parallel mode of depolymerization is faster than a serial mode of depolymerization. Here, we computationally implement severing process by segmenting single actin filament to multiple actin filaments with a length of 300 nm. Then, the lengths of segmented actin filaments are computed with the equation, $\frac{{dL_{i,1}^{A0} }}{dt} = - v_{s}$ at the pointed-end of actin filament where $v_{s}$ is shrinkage rate of 10 nm/s. We assume that severing duration time is set to be 20 s.

### Dynamic state change of F-actin

During the dynamic process, the state of single actin filament changes depending on the adhesion of barbed-end for actin filament to the actin cortex layer (ACL). This can be modelled as a discrete state transition network detailed in Supplementary Fig. 2. Briefly, a daughter actin filament is nucleated at Arp2/3 complexes on a mother actin filament with a nucleated rate (*k*_*n*_) of 50 s^−1^ (nucleated state, S = 1), then the daughter actin filament grows with a directional angle of 70 degrees from the mother actin filament (growth state, S = 2) until the barbed-end for actin filament contact to the surface of the actin cortex layer. In addition, the formation of adhesions of actin filaments to the surface of ACL is assumed to occur when *h*_*p*_ (gap between the barbed-end for actin filament and the surface of ACL) is less than 100 nm (adhesion state, S = 3). This adhesion is also assumed to rupture when tensile force is higher than 20 pN (detach state, S = 4) due to Brownian ratchet motion at the cellular membrane, and then immediately actin polymerization occurs (ratchet state, S = 5). In particular, it is known that increasing the capping protein (CP) concentration decrease the density of actin network, while increasing the Arp2/3 complex concentration increases the network density^33^. Accordingly, during invadopodia retraction and severing phases, all the barbed-ends for actin filaments at the tip of invadopodia are forced to bind with CPs to block the actin polymerization (capping state, S = 6). For the rest of leading edge of the cell, CPs are assumed to bind to barbed-ends for actin filaments (growth or ratchet state) with a binding rate (*k*_*cap*_) of 0.3 s^−1^. Lastly, CPs are uncapped to resume actin polymerization when invadopodia protrusion phase is recycled (uncapping state, S = 7).

### Characterization of invadopodia protrusion

First, three series of simulations were performed to investigate the effects of protrusive phase on the protrusion of invadopodia into ECM by changing time durations of protrusive phase (60, 120, and 240 s) (Fig. 4a,b). Here, time durations in retractile and severing phases for each case were kept constant at 60 and 20 s, respectively. The time duration in the retractile phase represents the turnover time of non-muscle myosin II. Computational model of branched actin network was constructed with two patterns of actin filaments. Initially, the first pattern of 500 actin filaments as a base case was randomly aligned so that its barbed-end and pointed-end for actin filaments are close to the inner cellular membrane and the nuclear membrane, respectively. Each actin filament can rapidly polymerize at the bared-end with a rate of 20 nm/s, depolymerize at the pointed-end with a rate of (10 nm/s)^23^, and Arp2/3 complex binds the mother filaments and initiates the polymerization of daughter filament at a branch angle of 70 degree from the mother filament. Then, the second pattern of 500 actin filaments was also randomly distributed to encircle the nuclear membrane, and a pair of these filaments were cross-linked by a bipolar myosin filament with an unstressed length of 300 nm and α-actinin with an unstressed length of 50 nm. Computational model of ECM fiber network (45 × 30 × 20 µm) is constructed with a pore size of 3.0 µm and single collagen type 1 fiber diameter of 41 nm. The presented computational method of ECM and its simulated results are consistent with recent cell migration experimental observations. Computational model successfully reproduced these observations that speeds of both tip and root of filopodia increase as the pore size the collagen gel is increased in experiments^21^. Previously, to characterize bulk modulus of ECM fiber network with a pore size of 3.0 µm, a simulation of mechanical stretching test for corresponding ECM fiber segment was performed, and bulk modulus of ECM model was found to be (2,558 Pa)^22^. Then, spherical cell model was initially placed in the center of ECM domain, and two kinds of volume exclusion effects of the cell were considered (*i*) to prevent ECM fibers from penetrating though external cellular membrane, and (*ii*) to keep F-actin in the intracellular domain with two surface boundaries of internal cellular membrane and nuclear membranes (Supplementary Fig. 3).

As a result, bipolar myosin filaments are densified and aligned along the surface of the nuclear membrane. Thereby, the nucleus was pulled toward to the branched actin filament network because strong contractile force was generated by densified bipolar myosin filaments (Fig. 4a and Supplementary Movie 1). In addition, we found that as the duration time in the protrusive phase increases, the formation of invadopodia protrusion was enhanced since MT1-MMP can be secreted to degrade ECM surrounding the cells (Fig. 4b and Supplementary Movie 2). We also found that the speed of nucleus was faster when the time duration in the retractile phase (or turnover time of non-muscle myosin II) is longer (Fig. 4c). Interestingly, multiple cycles of invadopodia elongation and retraction is captured for all three cases (Fig. 4d), reproducing the experimental observation^12^. The simulated migrated distance of invadopodial tip showed an excellent linear correlation to the duration time of protrusive phase (Fig. 4e).

Second, three series of simulations were performed and compared to investigate the effect of initial number of F-actin (1000, 2000, and 3000) on the invadopodia protrusion (Supplementary Fig. 4). In these simulations, time durations in the protrusive, retractile, and severing phases were kept constant at 240, 60, and 20 s. At these parameters, the speed of invadopodia was highest according to the first set of simulations. At time-point of 900 s, simulated plots of all three cases shows sharp invadopodia protrusions with densified branched actin network into ECM fiber network (Supplementary Fig. 4a–c). Hence, both the number of adhered F-actin to the cellular membrane and the speed of invadopodium increase as the number of initial F-actin increases (Supplementary Fig. 4d,e). Statistical analysis of linear regression was performed by comparing the number of adhered F-actin and the speed of invadopodium in terms of the number of initial F-actin. Excellent correlations were found between the two with r^2^ = 0.998 (Supplementary Fig. 4f.).

### Experimental observation of invadopodia protrusion and MT1-MMP inhibition.

We elect to test the validity of our computational model by observing the dynamics of invadopodia formation of cancer cells cultured within a 3D collagen I extracellular matrix (ECM). We chose MDA-MB-231 breast carcinoma cells in our study as they were widely reported to produce invadopodia when cultured in ECM^34–36^. To observe the interaction of the invadopodia with the collagen type I ECM, we perform live-cell time-lapse confocal microscopy imaging of cancer cells cultured in the microfluidic device. MDA-MB-231 cancer cells cultured in collagen type I ECM are elongated and produce invadopodia to probe the surrounding matrix. The time-lapse images of the invadopodia reveal that they are incredibly active, and cancer cells use invadopodia to probe the surrounding matrix (Supplementary Movie 3). To quantify the invadopodia protrusion dynamics, the movement of invadopodial tip from each cell was tracked, and the average speed of this movement was calculated to be 22.5 nm/s.

**Figure 5:**
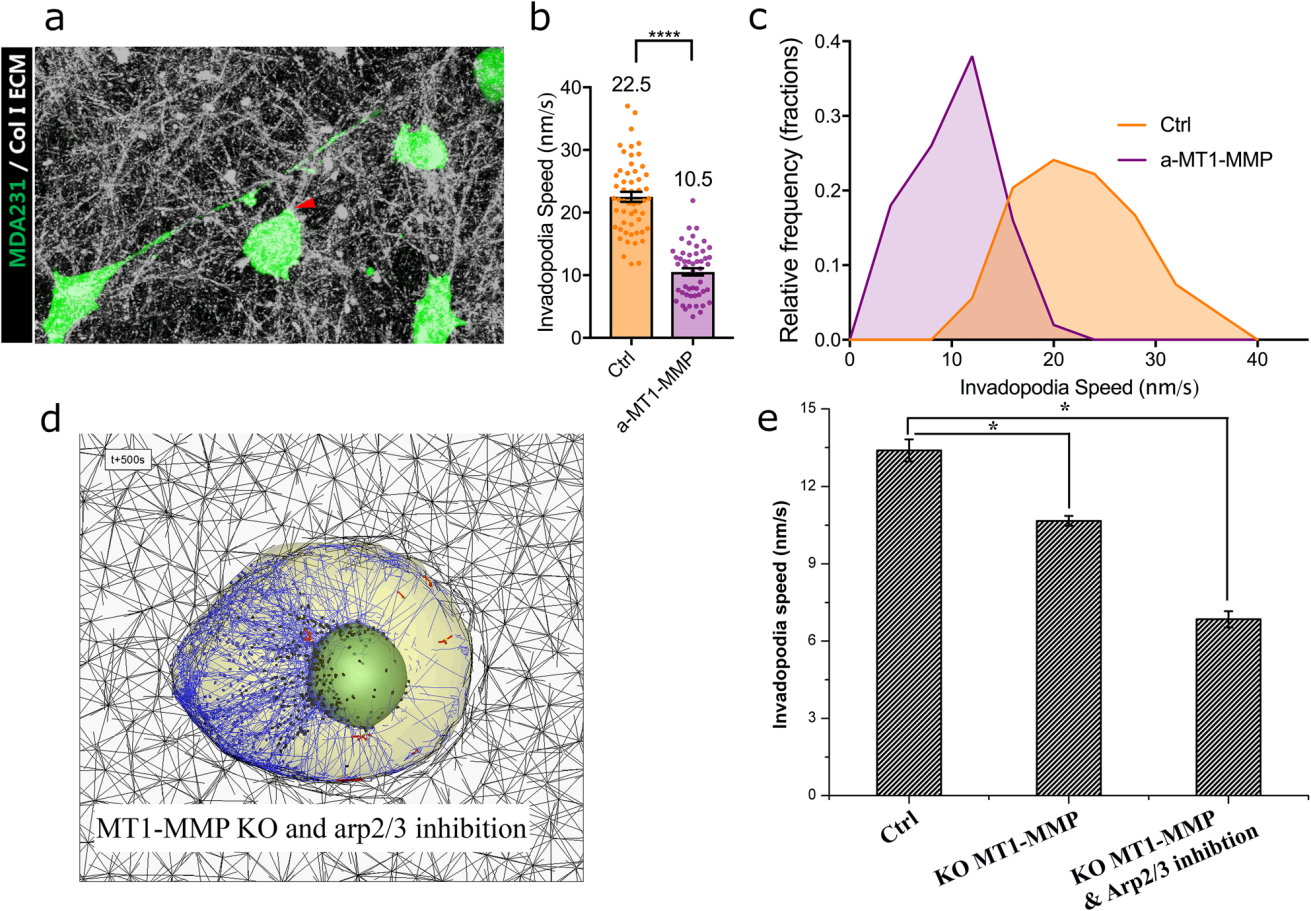
Experimental observation of invadopodia mechanosensing. GFP expressing MDA-MB-231 (MDA231) cells were cultured within a collagen I ECM, and the movement of the invadopodia were imaged and quantified by confocal time-lapse microscopy. (**a**) MDA231 (green) cells cultured in collagen I ECM (white) sends out elongated invadopodia (red arrows) to probe the ECM (scale bar = 30 μm). Quantification (**b**), mean; (**c**), frequency distribution) of the invadopodia speed from cells treated with antibody against MT1-MMP (a-MT1-MMP) and cells treated with IgG control (Ctrl). For (**b**), data shown are means (displayed above the bar) ± s.e.m from invadopodia from n = 50–54 cells. ****, p < 0.0001 from unpaired student t-test. (**d**) A selected still shot of simulated cancer cell with MT1-MMP knockout and Arp2/3 inhibition in ECM fiber network at the time-point of 500 s. (**e**) Bar graphs showing time-averaged speeds and s.e.m at the tip of invadopodium for three different cases of Ctrl, KO MT1-MMP only, and KO MT1-MMP and Arp2/3 inhibition. [*P < 0.05, n = 12, 11, 11, one-way ANOVA, posthoc Tukey’s test].

To evaluate our in-silico model, we incubated MDA-MB-231 cancer cells with blocking antibody against MT1-MMP, and quantified the invadopodia movement speed as described before. Inhibition of MT1-MMP activities by blocking antibody significantly reduced the activity of the invadopodia (red arrow in Fig. 5a, and Supplementary Movie 4) and the average invadopodia speed (from 22.5 nm/s to 10.5 nm/s, Fig. 5b). Moreover, the distribution of the invadopodia speeds shifts toward lower values as the result of MT1-MMP inhibition (Fig. 5c). These results match well with the outcome of our computational model with MT1-MMP knockout and the inhibition of Arp2/3 complexes (Fig. 5d and Supplementary Movie 5), indicating more significant reduction of the average invadopodia speed at this model (13.4 nm/s to 6.8 nm/s, Fig. 5e) than that of a model with MT1-MMP knockout only.

### Cancer cell invasive models with knockout of α-actinin, and filamin and fascin.

**Figure 6:**
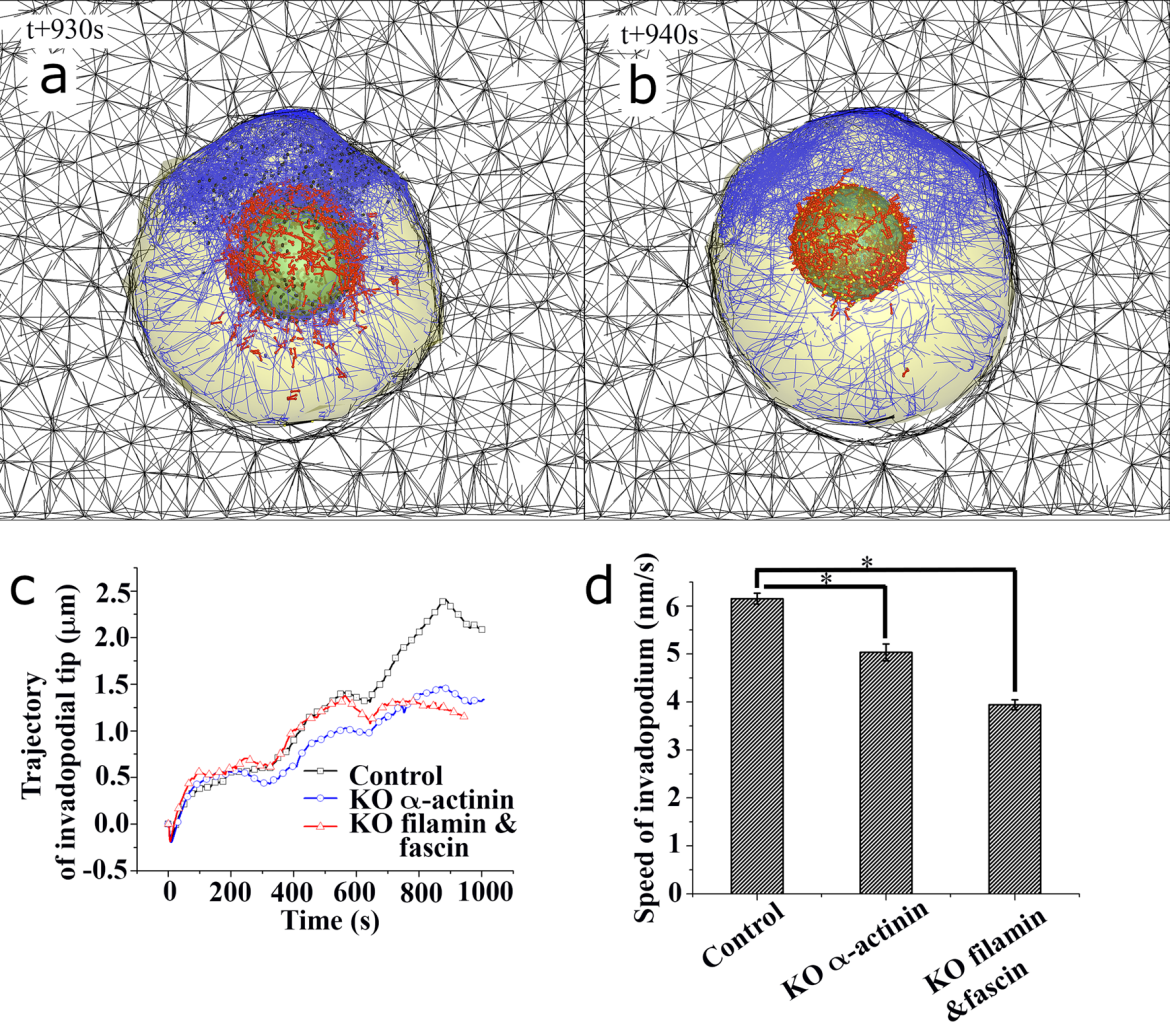
Cancer cell simulations with knockout (KO) of actin-crosslinking molecules. Selected plots of simulation for invadopodia protrusion in ECM with two cases of (**a**) KO α-actinin, and (**b**) KO filamin and fascin. Blue, red, yellow, dark blue, and black lines indicate F-actin, bipolar myosin filament, α-actinin, filamin and fascin, repectively. (**c**) Trajectory of invadopodial tip about three cases of control, KO α-actinin, and KO filamin and fascin. (**d**) Bar graphs showing time-averaged speeds and s.e.m at the tip of invadopodium for three different cases of control, KO α-actinin, and KO filamin and fascin. [*P < 0.05, n = 9, 9, 9, one-way ANOVA, posthoc Tukey’s test].

It has been known that overexpression of filamin A has a tumor-promoting effect when it is localized to the cytoplasm^37^. In addition, fascin up-regulation in the invadopodia is highly related to increased cancer cell motility^38^, and increased α-actinin-1 level promotes cell migration, especially in human breast cancers^39^. On the other hand, fascin elimination decreases migration in glioma cells^40^ and downregulations of both α-actinin-1 and α-actinin-4 make critical and distinct contributions to cytoskeletal organization, rigidity-sensing, and motility of glioma cells^41^. Therefore, cancer cells with impaired α-actinin, filamin, and fascin may have a reduction in motility. To investigate how α-actinin, filamin, and fascin affect cancer cell invasion (Fig. 6a,b), we computationally knocked out these proteins in our simulation and observed the resulting effects on invadopodia protrusion into ECM. Myosin filaments were observed to scatter out of the nucleus (Fig. 6a), which resulted in weak contractile force transmitted to both invadopodial and nuclear membranes (Supplementary Movie 6). Interestingly, invadopodia formation was significantly inhibited (Fig. 6b and Supplementary Movie 7), which resulted from the unbundling of actin filaments. Hence, knockouts of filamin and fascin significantly reduced the activity of the invadopodia (Fig. 6c,d) and the averaged invadopodia speed.

### Time evolutions of extracellular and intracellular forces during the directed cell migration.

**Figure 7:**
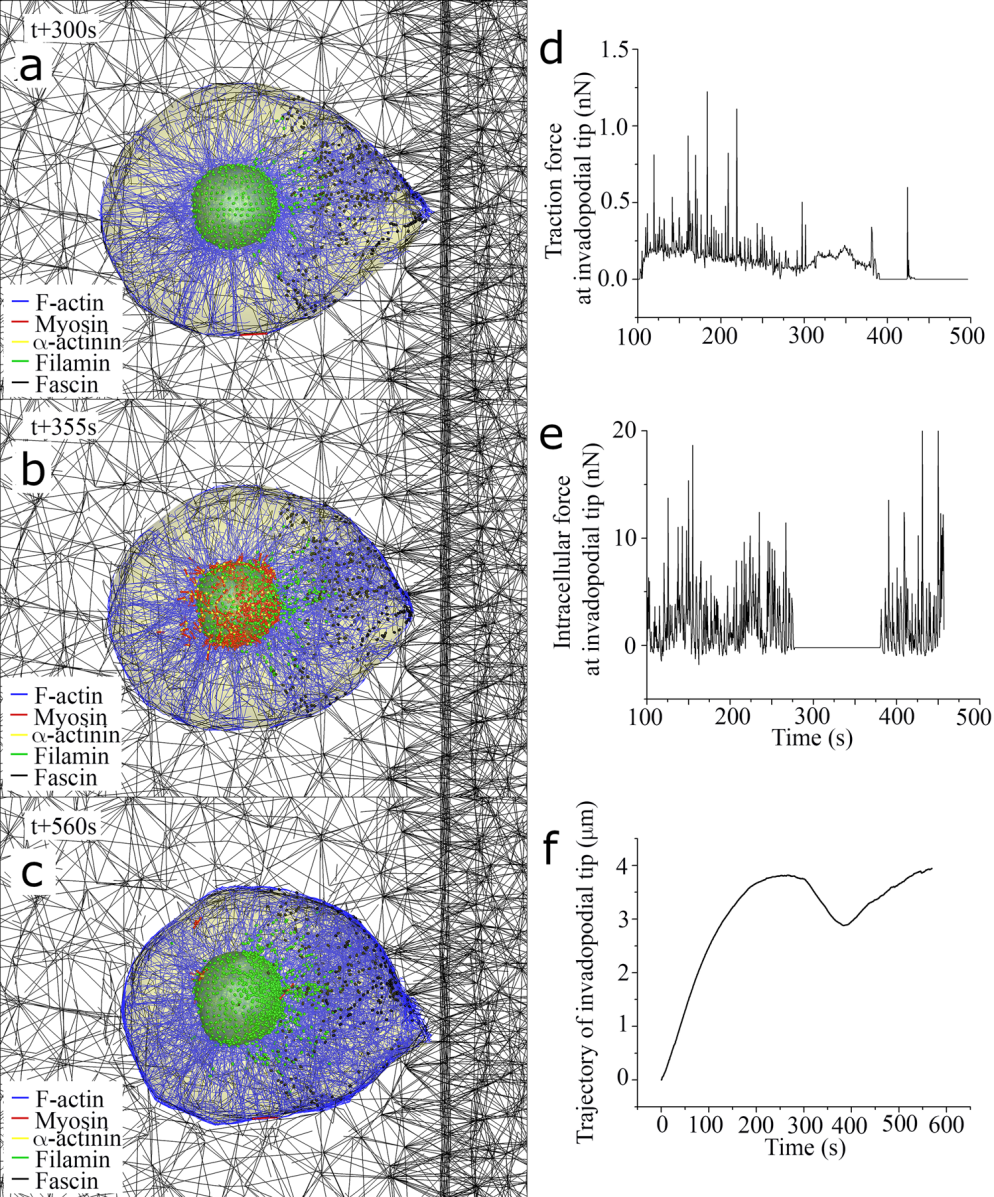
Cyclic motion of invadopodia dynamics during the directed cell migration towards stiffer ECM. Selected still shots of simulated cell migration toward stiffer ECM at time points of (**a**) 300 s (at the end of 1^st^ protrusive phase), (**b**) 355 s (at the end of 1st retractile phase), and (**c**) 560 s (at the end of 2^nd^ protrusive phase). Three graphs in (**d**), (**e**), and (**f**) show time-varying traction (extracellular) force, intracellular force, and trajectory at the tip of invadopodium, respectively.

Comparing the series of simulated data of actin filaments and invadopodia provides insights into the time evolution of traction and intracellular forces during the directed cell migration toward stiffer ECM. To analyze dynamic behavior of invadopodium during stiffness-directed cell migration, the simulation was set up with cells located 10 µm from the sharp interface between soft and stiff ECMs. In this case, time durations of protrusive, retractile, and severing phases were set to be 300, 60, and 20 s, respectively. It has recently been reported that the secretion rate of MT1-MMP at the tip of invadopodium is enhanced in a stiffer ECM^13,42^. Accordingly, in this simulation, the secretion rate of MT1-MMP at the tip of invadopodium is assumed to be positively correlated with the density of surrounding ligand. As shown in Fig. 7a,c, three selected plots of branched actin network and ECM fiber network at time points of 300 s, 355 s, and 560 s show how branched actin filaments are gradually densified as the tip of invadopodium approaches stiffer ECM (Supplementary Movie 8). Temporal variations of traction force (Fig. 7d), intracellular force (Fig. 7e), and trajectory (Fig. 7f) at the tip of invadopodium were evaluated in the time range of 100–500 s. Time-averaged traction force and intracellular force were calculated as 0.10 nN and 1.16 nN, respectively. In Fig. 7f, the trajectory of invadopodial tip shows multiple cycles of invadopodia elongation and retraction, with maximum at 260 s and minimum at 380 s. Unlike the first cycle, protrusive distance of invadopodial tip at the second cycle was reduced because of sharp interface between soft and stiff ECMs, which indicates significant increase in traction force at the tip of invadopodium.

In this report, our simulated data reveal that contractile forces, adjusted by the turnover time of perinuclear myosin filaments, can induce the movement of nucleus, which can facilitate the cancer cell invasion and migration into extracellular matrix confinement. Interestingly, a recent study revealed that non-muscle myosin II filaments have fast turnover with a half time of 1 min, while the half-life in skeletal muscle cells is more than an hour^43^. Accordingly, we reflect this fast turnover time into our model, and found that the fast turnover induces nuclear movement towards the branched actin network. As for the nuclear movement, it has been reported that the formation of perinuclear actin fibers induces temporal rotational movement of the nucleus, which results in nuclear reorientation to the direction of migration^44^. Further, our computational model was able to predict the role of actin crosslinkers in invadopodia formation by computationally depleting α-actinin, filamin, and fascin, from the branched actin network. For example, through a simulation of α-actinin depletion, we reveal that bipolar myosin filaments are impossible to generate contractile forces without α-actinin, resulting in impaired nuclear movement. In addition, in case of a simulation for the knockout of filamin and fascin, we also found that invadopodial membrane cannot protrude without bundling of actin filaments by filamin and fascin.

We introduce a computational framework for simulating the invasion of metastatic cancer cell into ECM by integrating mechanics of intracellular branched actin filaments and discrete ECM fibers in the invadopodia protrusion dynamics. This computational framework includes two-way interactions between intracellular and extracellular domains. The effects of intracellular domain on extracellular one are represented by the remodeling and densification of ECM via mechanical force generated by invadopodia and chemical degradation of cross-linked ECM fibers. The effects of extracellular domain on intracellular one are modeled as the adaptation of branched actin network to the stiffness and density of surrounding ECM. In addition, it is known that a fluid-to-solid transition of cells occurs in epithelial tissues when a dimensionless parameter, $q = {p \mathord{\left/ {\vphantom {p {\sqrt A }}} \right. \kern-\nulldelimiterspace} {\sqrt A }} \propto \frac{{F_{cell - cell} }}{{F_{actin\,cortex} }}$ (where *p* is perimeter of single cell, *A* is the area of the cell), is larger than 3.81, which represents fluid-like tissue or unjammed cells of disordered metastable tissue configurations^45^. For example, the cell with many filopodia behaves like fluid since its perimeter is larger. However, in our case, we assume that invadopodia protrusion has increased actin branching density than that of filopodia protrusion^46^.

MT1-MMP accumulates at tips of invadopodia and plays a crucial role in focal pericellular degradation of the BM^47^. Furthermore, it has been proposed that high invasiveness of cancer cell migration occurs at the intermediated levels of ECM crosslinking (0.39) which strictly depends on proteolysis of the matrix by MT1-MMP^48^. It is known that the activation of integrins leads to the assembly of F-actin, Arp2/3 complex, formins, and cortactin in the invadopodia^49^. Aggregation of F-actin and cortactin initiates the accumulation of MT1-MMP at the invadopodia. F-actin density and MT1-MMP are related because experimental findings show that depletion of the protease impairs F-actin and cortactin accumulation at the invadopodial tip^50^. In particular, cortactin is one of key components involved in the delivery of MT1-MMP to invadopodia, and cortactin has an important role as a regulator of arp2/3-mediated actin branching^51^. Furthermore, Arp2/3 overexpression is tightly associated with cancer progression and tumor cell invasion. Arp2/3 overexpression was also associated with poor patient survival in lung^52^ and breast cancers^53^. Arp2/3 overexpression in breast cancer is associated with HER2 overexpression^54^. Based on above experimental findings, our simulations suggest that Arp2/3 is involved with the secretion of MT1-MMP, which is not necessarily verified. Accordingly, we reflect this correlation between Arp2/3 mediated actin branching and the secretion of MT1-MMP in our model to match our computational simulation with experimental results of MT1-MMP knockout. Thereby, we found more significant reduction in average invadopodia speed using our current model, which considers both MT1-MMP chemical signaling and Arp2/3-mediated mechanical branching of actin (Fig. 5e), than a model that only considers MT1-MMP signaling.

Current model for the invadopodia protrusion is inspired by above-mentioned experimental findings. We assume that single F-actin can induce Arp2/3-mediated actin branching when this F-actin is connected to the activated integrins through the force transduction layer. Subsequently, actin branching is further densified at the proximal invadopodium, which results in the recruitment of more activated integrin clustering and strong traction force and stress over the surrounding ECM. Recently, an interesting finding is the adaptation of branched actin network, that is, actin branching become denser when higher external force is exerted on actin network^55^. Thereby, as the surrounding ECM is stiffer and denser, the size of focal adhesions is larger, which will result in denser branched actin network. Eventually, cell needs to modulate to high secretion rate of MT1-MMP. In this aspect, the two-way interactions between cell (actin) and ECM can be coupled and feed-backed each other to promote invadopodia activity.

We note that our model of the invadopodia protrusion into ECM fiber network is complex and multifaceted. This model takes into account of impacts ECM stiffness and density on branched actin network, initial concentrations of actin and crosslinking molecules, rates of actin polymerization and depolymerization at the barbed-ends and pointed-ends for actin filaments, turnover time of mechanosensitive contractile bipolar myosin filament, and binding rate of CP at the barbed-end for actin filaments. Among them, the most sensitive parameter is the turnover time of myosin since turnover time > 90 s results in strong contractile force that pulls the nucleus too close to the invadopodial membrane. A recent study found that a rigid ECM promotes MT1-MMP secretion at the invadopodia^13^. Importantly, both the adaptation of branched actin network and MT1-MMP secretion rate are controlled by the surrounding ECM stiffness, which represents invadopodia mechanosensing. Current computational model is limited in feedback control of MT1-MMP secretion by cell-ECM interactions. Therefore, future invadopodia model will need to incorporate this invadopodia mechanosensing. In addition, current model is also limited in the characterization and validation of single actin network model only, for example, the dependence of viscoelastic properties of actin network on crosslinker concentration and myosin activity. Instead, we looked into whether dynamic behaviors of invadopodia depend on myosin activity or actin crosslinking molecules by depleting these molecules in our simulations. To our knowledge, this is the first 3D model that predicts the experimentally observed invadopodia behaviors in respond to the inhibition of actin-crosslinking molecules. In addition, we focused on the validation of whole comprehensive model of cell-actin-ECM because it would be better to provide an insight of the model. Therefore, the extension of our current model will be to simulate how actin network will respond to indentation by deflectable cantilever and to predict the experimental results obtained via atomic force microscopy measurements^56,57^. Our current membrane model has fixed topologies and has a limitation in forming very narrow protrusions like filopodia. To overcome this limitation, we previously constructed filopodia geometric model by allowing the growth of filopodium patches with six triangular elements (or a hexagonal patch) on the cellular membrane^21,22^. These triangular elements were remeshed and new elements were allowed to generate along to the filopodium shaft. Interestingly, there has been a novel grand canonical membrane simulation model that describes stochastic polymerization of filaments against a fluctuating fluid membrane^58^. Their results suggest a membrane mediated dynamical transition from single filaments to cooperatively growing bundles as an important dynamical bottleneck to tubular protrusion. Finally, our ultimate goal is to use the computational model to understand the role that invadopodia protrusions play in cancer cell invasion and metastasis. To achieve this, the future direction of this model will need to simulate cancer cell extravasation and subsequent invasion into thin but dense BM along the blood vessels.

In summary, there have been few comprehensive models of invadopodia protrusion and invadopodia-ECM interactions due to the complexity of both the branched actin network and ECM fiber network. Moreover, how invadopodia speeds are related to the frequency of invadopodia protrusion is also poorly understood. Using the computational model we proposed in this study, we found that invadopodia speeds become faster as the protrusive phase becomes longer. In addition, using this computational model to simulate the knockout of actin-crosslinking molecules, we evaluated the effects of α-actinin on perinuclear bipolar myosin filaments, and filamin and fascin on branched actin network. Specifically, we showed that 1) it is impossible to generate contractile force without α-actinin, resulting in impaired nuclear movement; and 2) bundling of actin filaments by filamin and fascin is required for the invadopodia protrusion. The computational model was then applied to test why non-muscle myosin has a fast turnover time. We found that myosin turnover time of 1 min is enough to produce stable movement of the nucleus towards the leading edge of the cell. Altogether, we believe that our computational model has the potentials to be a critical tool in understanding cancer invasion and metastasis in various ECM microenvironments.

## Methods

### Computational model of invadopodia protrusion dynamics

The elastic force at the *i*-th node of invadopodia membrane (module CI in Table 1), ${\varvec{F}}_{E,i}^{I}$, is derived by using the virtual work theory in structural mechanics. To this end, the total elastic energy stored in the invadopodial membrane is obtained. Two types of total elastic energies are considered. One is the total elastic energy associated with distance changes between the nodes^59,60^:

15 $$ H_{L}^{I} = \frac{{\kappa_{L}^{I} }}{2}\sum\limits_{i = 1}^{line} {\left( {L_{i}^{I} - L_{i}^{I0} } \right)^{2} } $$

where $L_{i}^{I}$ is the length of the *i*-th line of the invadopodial membrane mesh and is updated at every time-step. $L_{i}^{I0}$ is its relaxed length. $\kappa_{L}^{I}$ is effective stiffness constants of the line elements of the invadopodial membrane (5.0 × 10^–5^ N/m)^61^. The other is the total elastic energy associated with area changes:

16 $$ H_{A}^{I} = \frac{{\kappa_{A}^{I} }}{2}\sum\limits_{i = 1}^{element} {\left( {\frac{{A_{i}^{I} - A_{i}^{I} }}{{A_{i}^{I0} }}} \right)}^{2} A_{i}^{I0} $$

where $A_{i}^{I}$ is the *i*-th mesh area of the invadopodial membrane and $A_{i}^{I0}$ is its relaxed values. $\kappa_{A}^{I}$ is an effective stiffness constant of area elements of the invadopodial membrane (1.0 × 10^–4^ N/m^2^)^61^. Then, ${\varvec{F}}_{E,i}^{I}$ can be obtained by differentiating these two kinds of total elastic energy,

17 $$ {\varvec{F}}_{E,i}^{I} = - \kappa_{L}^{I} \sum\limits_{i = 1}^{line} {\left( {L_{i}^{I} - L_{i}^{I0} } \right)} \frac{{\partial L_{i}^{I} }}{{\partial {\varvec{x}}_{i}^{I} }} - \kappa_{A}^{I} \sum\limits_{i = 1}^{element} {\left( {\frac{{A_{i}^{I} - A_{i}^{I0} }}{{A_{i}^{I0} }}} \right)} \frac{{\partial A_{i}^{I} }}{{\partial {\varvec{x}}_{i}^{I} }}. $$

The focal complex force at the *i*-th node of invadopodia membrane,${\varvec{F}}_{FC,i}^{I}$,is expressed as

18 $$ {\varvec{F}}_{FC,i}^{I} = n_{b,i}^{I} \kappa_{LR} \left( {L_{b} - \lambda } \right)\hat{\user2{n}}_{R,i}^{I} $$

where $n_{b,i}^{I}$ is the number of integrin-collagen bonds at the *i*-th node of invadopodial membrane,$\kappa_{LR}$ is the spring constant of a single integrin-collagen bond (~ 1 pN/nm)^62^, $L_{b}$ is the average stretched length of the integrin-collagen bonds, $\lambda$ is an unstressed length of bonds (~ 30 nm)^63^ and $\hat{\user2{n}}_{R,i}^{I}$ is a unit vector at the local surface of the *i*-th node of invadopodial membrane toward the bonding site at the ECM fiber. Here $\left( {L_{b} - \lambda } \right)$ represents the stretched distance from the equilibrium. We utilize Bell’s model^64^ to incorporate force-dependent reaction rates of $n_{b,i}^{I}$ is expressed in following ordinary differential equation:

19 $$ \frac{{dn_{b,i}^{I} }}{dt} = k_{on}^{{}} \left( {n_{tot}^{I} - n_{b,i}^{I} } \right) - k_{off}^{{}} n_{b,i}^{I} $$

where $n_{tot}^{I}$ is total available number of integrin molecules at the *i*-th node of invadopodial membrane, $k_{on}^{{}}$ is the kinetic associate rate for binding integrin molecules and ECM fiber, and it is expressed as^65,66^

20 $$ k_{on}^{{}} = k_{on}^{0} \frac{{l_{bind} }}{{Z_{0} }}\exp \left[ { - \frac{{\kappa_{LR} \left( {L_{b} - \lambda } \right)^{2} }}{{2k_{b} T}}} \right] $$

where $k_{on}^{0}$ is the zero forward reaction rate (1 molecule^−1^ s^−1^), $l_{bind}$ is a binding radius (30 nm) to check whether the *i*-th node of invadopodial membrane and the node on the fiber are sufficiently close, and $k_{b} T$ is the unit of thermal energy.$Z_{0}$ is the partition function for a integrin molecule confined in a harmonic potential between $- \lambda$ and $L_{b} - \lambda$, and it is expressed as

21 $$ Z_{0} = \sqrt {\frac{{\pi k_{b} T}}{{2\kappa_{LR} }}} \left( {erf\left[ {\left( {L_{b} - \lambda } \right)\sqrt {\frac{{\kappa_{LR} }}{{2k_{b} T}}} } \right] + erf\left[ {\lambda \sqrt {\frac{{\kappa_{LR} }}{{2k_{b} T}}} } \right]} \right). $$

$k_{off}^{{}}$ is the kinetic dissociation rate, and it is known as Bell’s equation for the slip bond, which is defined by^64^

22 $$ k_{off} = k_{off}^{0} \exp \left[ {\frac{{\kappa_{LR} \left( {L_{b} - \lambda } \right)x_{b} }}{{k_{b} T\,}}} \right] $$

where $$k_{off}^{0}$$ is the zero kinetic dissociation rate in the absent of the force, $$x_{b}$$ is the distance between the minimum binding potential and the transition state barrier, and $${\raise0.7ex\hbox{${k_{b} T}$} \!\mathord{\left/ {\vphantom {{k_{b} T} {x_{b} }}}\right.\kern-\nulldelimiterspace} \!\lower0.7ex\hbox{${x_{b} }$}}$$ represents an intrinsic force ~ 200 pN. The transduce force at the *i*-th node of invadopodia membrane,$${\varvec{F}}_{T,i}^{I}$$, is expressed as

23 $$ {\varvec{F}}_{T,i}^{I} = - \kappa_{T} \left( {L_{T,i}^{{}} - L_{T,i}^{0} } \right)\frac{{\partial L_{T,i}^{{}} }}{{\partial {\varvec{x}}_{i}^{I} }} $$

where $\kappa_{T}$ is an effective spring constant of line element of the FTL (8.0 × 10^–3^ N/m), $L_{T,i}^{{}}$ is the tensioned length of the *i*-th line in the FTL, and it is updated at every time-step. $L_{T,i}^{0}$ is its relaxed (zero force) length (50 nm).

The elastic force at the *i*-th node of force transduce layer (FTL) (module CT in Table 1), ${\varvec{F}}_{E,i}^{T}$, is expressed as

24 $$ {\varvec{F}}_{E,i}^{T} = - \kappa_{L}^{T} \sum\limits_{i = 1}^{line} {\left( {L_{i}^{T} - L_{i}^{T0} } \right)} \frac{{\partial L_{i}^{T} }}{{\partial {\varvec{x}}_{i}^{T} }} $$

where $\kappa_{L}^{T}$ is an effective spring constant of line element of the FTL (8.0 × 10^–3^ N/m), $L_{i}^{T}$ is the stressed length of the *i*-th line on the FTL mesh and is updated at every time-step. $L_{i}^{T0}$ is its relaxed length. The actin cortex force the *i*-th node of FTL,${\varvec{F}}_{C,i}^{T}$, is expressed as

25 $$ {\varvec{F}}_{C,i}^{T} = - \kappa_{C} \left( {L_{C,i}^{{}} - L_{C,i}^{0} } \right)\frac{{\partial L_{C,i}^{T} }}{{\partial {\varvec{x}}_{i}^{T} }} $$

where $\kappa_{C}^{{}}$ is an effective spring constant of line element of the actin cortex layer (ACL) (1.0 × 10^–2^ N/m), $L_{C,i}^{{}}$ is the tensioned length of the *i*-th line in ACL mesh, and it is updated at every time-step. $L_{C,i}^{0}$ is its relaxed (zero force) length (50 nm).

The elastic force at the *i*-th node of ACL (module CC in Table 1),${\varvec{F}}_{E,i}^{ACL}$, is expressed as

26 $$ {\varvec{F}}_{E,i}^{C} = - \sum\limits_{i = 1}^{line} {\frac{{A_{A} E_{A} }}{{L_{i}^{C0} }}\left( {L_{i}^{C} - L_{i}^{C0} } \right)} \frac{{\partial L_{i}^{C} }}{{\partial {\varvec{x}}_{i}^{C} }} $$

where $A_{A}$ is the sectional area of the actin filament ($\uppi {r}_{A}^{2}$), where $r_{A}^{{}}$ is the radius of actin filament (3.5 nm), and $E_{A}$ is Young’s modulus of the actin filament, $L_{i}^{C}$ is the length of the *i*-th line on the ACL mesh and is updated at every time-step. $L_{i}^{C0}$ is its relaxed length.

In a similar manner of the invadopodia membrane, the elastic force at the *i*-th nuclear membrane (module CN in Table 1),${\varvec{F}}_{E,i}^{N}$ , is obtained by using the virtual work theory in structural mechanics. Two types of total elastic energy are considered in the nuclear membrane. Then, ${\varvec{F}}_{E,i}^{N}$ can be obtained by differentiating the two kinds of total energy,

27 $$ {\varvec{F}}_{E,i}^{N} = - \kappa_{L}^{N} \sum\limits_{i = 1}^{line} {\left( {L_{i}^{N} - L_{i}^{N0} } \right)} \frac{{\partial L_{i}^{N} }}{{\partial {\varvec{x}}_{i}^{N} }} - \kappa_{A}^{N} \sum\limits_{i = 1}^{element} {\left( {\frac{{A_{i}^{N} - A_{i}^{N0} }}{{A_{i}^{N0} }}} \right)} \frac{{\partial A_{i}^{N} }}{{\partial {\varvec{x}}_{i}^{N} }}. $$

where $L_{i}^{N}$ is the length of the *i*-th line of the nuclear membrane mesh and is updated at every time-step. $L_{i}^{N0}$ is its relaxed length. $\kappa_{L}^{N}$ is effective stiffness constants of the line elements of the nuclear membrane (5.0 × 10^–3^ N/m)^59,60^. $A_{i}^{N}$ is the *i*-th mesh area of the nuclear membrane and $A_{i}^{N0}$ is its relaxed values. $\kappa_{A}^{N}$ is an effective stiffness constant of area elements of the nuclear membrane (1.0 × 10^–4^ N/m^2^)^61^. ${\varvec{F}}_{L,i}^{N}$ is the actin crosslinking force by the *l*-th filamin or α-actinin which is connected to the *i*-th node in the nuclear membrane, and it is expressed as

28 $$ {\varvec{F}}_{L,i}^{N} = \kappa_{L}^{ACL} (L_{l}^{ACL} - L_{l}^{ACL0} )\frac{{\partial L_{l}^{ACL} }}{{\partial {\varvec{x}}_{k,l}^{AF} }}. $$

### Computation of viscoelastic cancer cell model

Cancer invadopodia protrusion simulations were carried out using a fourth order Rosenbrock method based on an adaptive time-stepping technique for integrating ordinary differential equations with the convergence criterion < 10^−4^. The ordinary differential equations of invadopodia membrane, FTL, ACL, and branched actin network, and double layers of nuclear membrane (PAL and NMS) were numerically coupled to solve for unknown variables associated with node position vectors for all of computational domains (see Table 1). Thereby, all of five submodules were coupled with viscoelastic kelvin-voigt model (Fig. 2b and 3). To solve five coupled ordinary differential equations in the five domains numerically, these equations should be converted with respect to vectors as followings:

29 $$\left[{\varvec{C}}\right]{\left\{\frac{d{{\varvec{x}}}^{I}}{dt},\frac{d{{\varvec{x}}}^{T}}{dt},\frac{d{{\varvec{x}}}^{C}}{dt},\frac{d{{\varvec{x}}}^{A}}{dt},\frac{d{{\varvec{x}}}^{N}}{dt}\right\}}^{T}={\left\{{{\varvec{F}}}^{I},{{\varvec{F}}}^{T},{{\varvec{F}}}^{C},{{\varvec{F}}}^{A},{{\varvec{F}}}^{N}\right\}}^{T}$$

30 $${\left\{\frac{d{{\varvec{x}}}^{I}}{dt},\frac{d{{\varvec{x}}}^{T}}{dt},\frac{d{{\varvec{x}}}^{C}}{dt},\frac{d{{\varvec{x}}}^{A}}{dt},\frac{d{{\varvec{x}}}^{N}}{dt}\right\}}^{T}={{\left[{\varvec{C}}\right]}^{-1}\left\{{{\varvec{F}}}^{I},{{\varvec{F}}}^{T},{{\varvec{F}}}^{C},{{\varvec{F}}}^{A},{{\varvec{F}}}^{N}\right\}}^{T}$$

where $\left[ {\varvec{C}} \right] \in {\mathbb{R}}^{{3N_{cell} \times 3N_{cell} }}$ is dissipation coefficients matrix (Table 1), $N_{cell} = N_{I} + N_{T} + N_{C} + N_{A} + N_{N}$. Instead of obtaining a solution of Eq. (29) implicitly, we solve Eq. (30) explicitly based two reasons: 1) implicitly solving Eq. (29) is expensive because matrix $\left[ {\varvec{C}} \right]^{{}}$ is required to update at every iteration as the geometric structure of branched actin network is time-varying, and 2) matrix $\left[ {\varvec{C}} \right]^{{}}$ becomes singular without the inclusion of viscous drag coefficients in Table 1. Thereby, explicit forms of five equations in the cell module can be expressed as followings:

31 $$ \frac{{d{\varvec{x}}_{i}^{I} }}{dt} = \frac{{\left( {{\varvec{F}}_{E,i}^{I} + {\varvec{F}}_{FC,i}^{I} + {\varvec{F}}_{T,i}^{I} + {\varvec{F}}_{D,i}^{I0} } \right)}}{{\sum\limits_{j = 1}^{{n_{j} }} {C_{i,j}^{I} } + C_{1}^{T} + C_{0}^{I} }},\,\,\,\,\,\,i = 1, \cdots ,N_{I} $$

32 $$ \frac{{d{\varvec{x}}_{i}^{T} }}{dt} = \frac{{\left( {{\varvec{F}}_{E,i}^{T} + {\varvec{F}}_{T,i}^{T} + {\varvec{F}}_{A,i}^{T} + {\varvec{F}}_{D,i}^{T0} } \right)}}{{\sum\limits_{j = 1}^{{n_{i} }} {C_{i,j}^{T} } + C_{1}^{T} + C_{1}^{C} + C_{0}^{T} }},\,\,\,\,\,\,i = 1, \cdots ,N_{T} $$

33 $$ \frac{{d{\varvec{x}}_{i}^{C} }}{dt} = \frac{{\left( {{\varvec{F}}_{E,i}^{C} + {\varvec{F}}_{A,i}^{C} + {\varvec{F}}_{P,i}^{C} + {\varvec{F}}_{D,i}^{C0} } \right)}}{{\sum\limits_{j = 1}^{{n_{j} }} {C_{i,j}^{C} } + C_{1}^{C} + C_{0}^{C} }},\,\,\,\,\,\,i = 1, \cdots ,N_{C} $$

34 $$ \frac{{d{\varvec{x}}_{i,j}^{A} }}{dt} = \frac{{\left( {{\varvec{F}}_{E,i,j}^{A} + {\varvec{F}}_{ARP,i,j}^{A} + {\varvec{F}}_{br,i,j}^{A} + {\varvec{F}}_{L,i,j}^{A} + {\varvec{F}}_{C,i,j}^{A} + {\varvec{F}}_{D,i,j}^{A0} } \right)}}{{2C_{i,j}^{A} + C_{i,ARP}^{A} + C_{0}^{A} }},\,\,\,\,\,\,i = 1, \cdots ,N_{A}^{{}} $$

35 $$ \frac{{d{\varvec{x}}_{i}^{N} }}{dt} = \frac{{\left( {{\varvec{F}}_{E,i}^{N} + {\varvec{F}}_{L,i}^{N} + {\varvec{F}}_{D,i}^{N0} } \right)}}{{\sum\limits_{j = 1}^{{n_{j} }} {C_{i,j}^{N} } + C_{i0}^{N} }},\,\,\,\,\,\,i = 1, \cdots ,N_{N} $$

where ${\varvec{F}}_{D,i}^{I0} = \sum\limits_{j = 1}^{{n_{j} }} {C_{i,j}^{I} \frac{{d{\varvec{x}}_{j}^{I0} }}{dt}} + C_{1}^{T} \frac{{d{\varvec{x}}_{i}^{T0} }}{dt}$ represents the dissipated force at neighboring nodes of the *i*-th node in invadopodia membrane at the previous time-step.${\varvec{F}}_{D,i}^{T0} = \sum\limits_{j = 1}^{{n_{i} }} {C_{i,j}^{T} \frac{{d{\varvec{x}}_{j}^{T0} }}{dt}} + C_{1}^{T} \frac{{d{\varvec{x}}_{i}^{I0} }}{dt} + C_{1}^{C} \frac{{d{\varvec{x}}_{i}^{C0} }}{dt}$ represents the dissipated force at neighboring nodes of the *i*-th node in the FTL at the previous time-step.${\varvec{F}}_{D,i}^{C0} = \sum\limits_{j = 1}^{{n_{i} }} {C_{i,j}^{C} \frac{{d{\varvec{x}}_{j}^{C0} }}{dt}} + C_{1}^{C} \frac{{d{\varvec{x}}_{i}^{T0} }}{dt}$ represents the dissipated force at neighboring nodes of the *i*-th node in the FTL at the previous time-step.${\varvec{F}}_{D,i,j}^{A0} = C_{i,j}^{A} \left( {\frac{{d{\varvec{x}}_{i,j - 1}^{A0} }}{dt} + \frac{{d{\varvec{x}}_{i,j + 1}^{A0} }}{dt}} \right) + C_{i,ARP}^{A} \frac{{d{\varvec{x}}_{k,1}^{A0} }}{dt}$ represents the dissipated force at neighboring nodes of the *j*-th node on the *i*-th actin filament at the previous time-step.${\varvec{F}}_{D,i}^{N0} = \sum\limits_{j = 1}^{{n_{i} }} {C_{i,j}^{N} \frac{{d{\varvec{x}}_{j}^{N0} }}{dt}}$ represents the dissipated force at neighboring nodes of the *i*-th node in the double layers of nuclear membrane (PAL and NMS) at the previous time-step.

### Discrete ECM dynamics and reaction–diffusion model

The detailed equations that govern discrete ECM dynamics and reaction–diffusion mass transfer are summarized in S1 Text^21,22^.

### Visualization of simulated results

All the images (Figs. 1, 2c,d, 4a,b, 5d, 6a,b, 7a,c, S1b, S3 and S4a-c) and supplementary movies (Movies 1, 2, 4, 5, 6, 7 and 8) were created using a commercial software, Tecplot 360 (version 2018 R2, https://www.tecplot.com/products/tecplot-360/).

### Cell culture and Preparation of Microfluidic Devices

GFP-expressing MDA-MB-231 breast carcinoma cells were cultured in DMEM. DMEM was supplemented with 10% FBS and 100 U/mL penicillin/streptomycin. Cells were grown in T25 flasks (Thermo Fisher Scientific) until 90% confluent. Then they were removed from the flask surface by incubation with trypsin/EDTA (Gibco) for 2 min, centrifuged, and then resuspended in a 2.5 mg/mL collagen I solution, before seeding into the 3D microfluidic devices.

### Preparation of Microfluidic Devices and Invadopodia Imaging

Preparation of 3D microfluidic devices, followed by cell seeding, was performed as previously described in detail^67^. In brief, microfluidic devices were prepared using polydimethylsiloxane (PDMS) and bonded to a glass coverslip. Microfluidic channels were coated with poly-D-lysine (PDL) (Sigma). Before injection of the gel solution into the devices, cells were mixed with the gel solution from the original stock. Following that, the central gel channel, in which invadopodia dynamics were later imaged, was filled with the collagen I solution, at a density of 2.5 mg/mL. Devices were then incubated for 30 min at 37 °C to polymerize. The concentration of NaOH in the collagen I solution was varied to allow accurate control of pH 8.0 during polymerization of the solution. Following the polymerization process, cancer cells were completely surrounded by collagen type I ECM with estimated pore size (1 μm)^68^. After overnight incubation, the microfluidic device was transferred to a confocal laser-scanning microscope (Zeiss) fitted with an environmental chamber operating at 37 °C and 5% CO_2_. Time-lapse microscopy was employed to record cancer cell movement in the 3D collagen I ECM. Protrusive and retractile invadopodia of the GFP-expressing HUVECs (green), and collagen fibers (confocal reflectance microscopy) were simultaneously imaged with a time interval of 3 min for 1.5 h.

## Supplementary Information

Supplementary Information 1.

Supplementary Video 1.

Supplementary Video 2.

Supplementary Video 3.

Supplementary Video 4.

Supplementary Video 5.

Supplementary Video 6.

Supplementary Video 7.

Supplementary Video 8.
