# Supplementary material for: A computational modeling of invadopodia protrusion into an extracellular matrix fiber network
Source: Sci Rep. 2022 Jan 24;12:1231. doi: 10.1038/s41598-022-05224-9 (PMC8786978; doi:10.1038/s41598-022-05224-9)
Supplement: Supplementary file 1 — Supplementary Information 1. [file 41598_2022_5224_MOESM1_ESM.docx]

**Supplementary Information**

**A computational modeling of invadopodia protrusion into an extracellular matrix fiber network**

**Min-Cheol Kim,^1^*** **Ran Li, ^2,3^ Rohan Abeyaratne, ^1^ Roger D. Kamm,^1,2^ and H. Harry Asada^1^***

^1^ Departments of Mechanical Engineering and ^2^Biological Engineering, Massachusetts Institute of Technology, Cambridge, MA 02139;^3^Center for Systems Biology, Massachusetts General Hospital Research Institute, Boston, MA 02114

* To whom correspondence should be addressed:

^★^e-mail: mincheol@mit.edu; asada@mit.edu.

**Supplementary Figures**


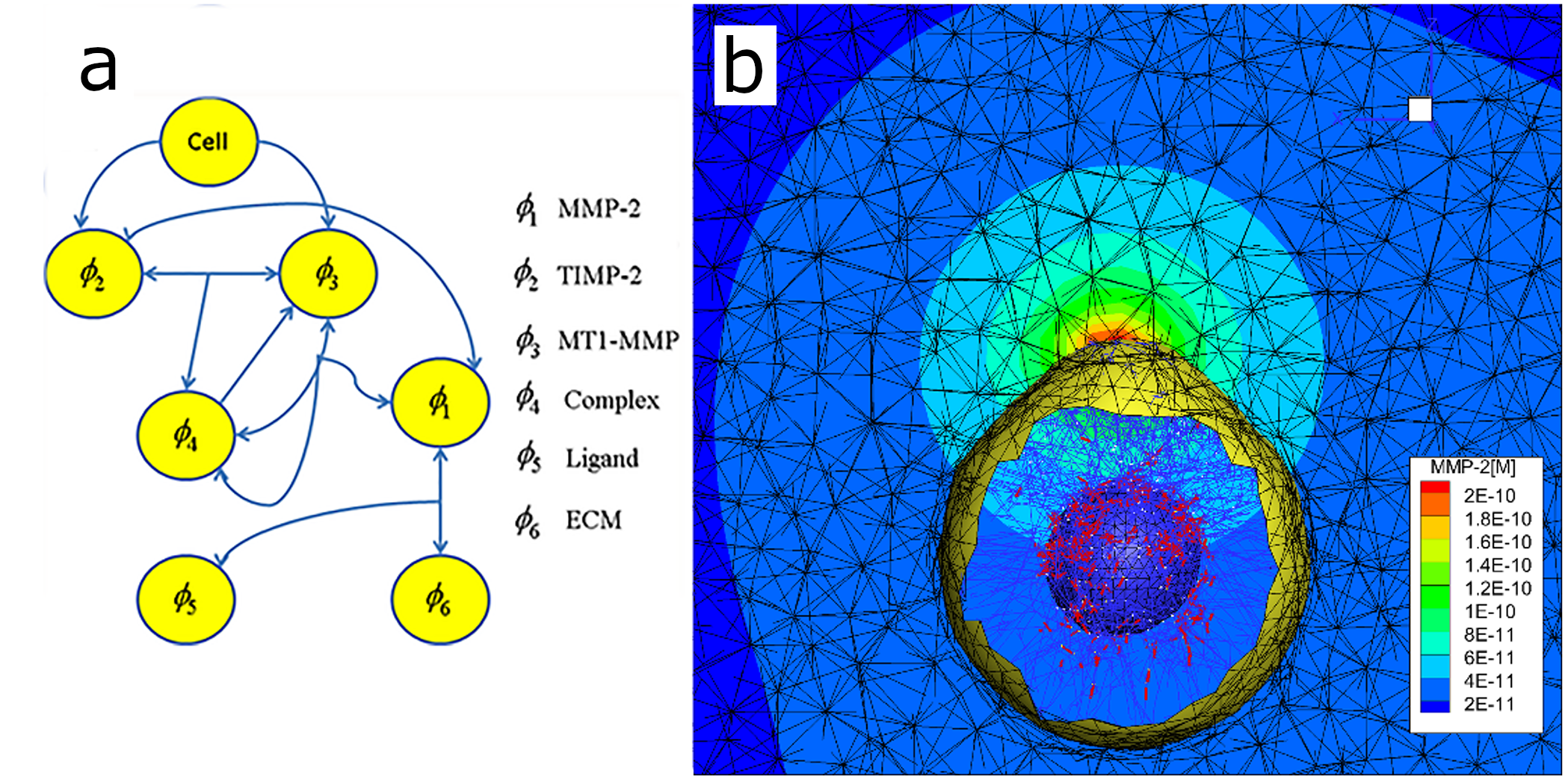


**Supplementary Figure 1**. Schematic diagram of signal pathway. **a**) The extracellular signal pathway activates MMP-2, and degrades the integrity of ECM. **b**) An example of simulated results showing MMP-2 concentration contour distribution over ECM fiber network model.


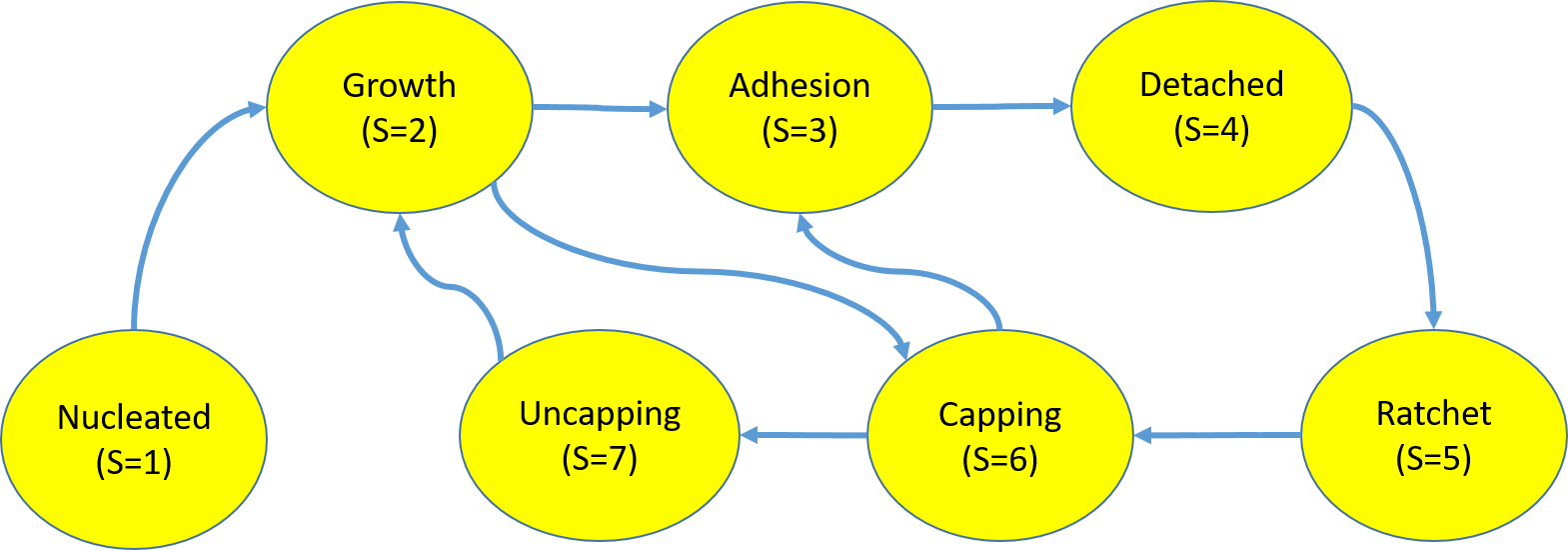


**Supplementary Figure 2**. Eight dynamic states at barbed-end of actin filament.


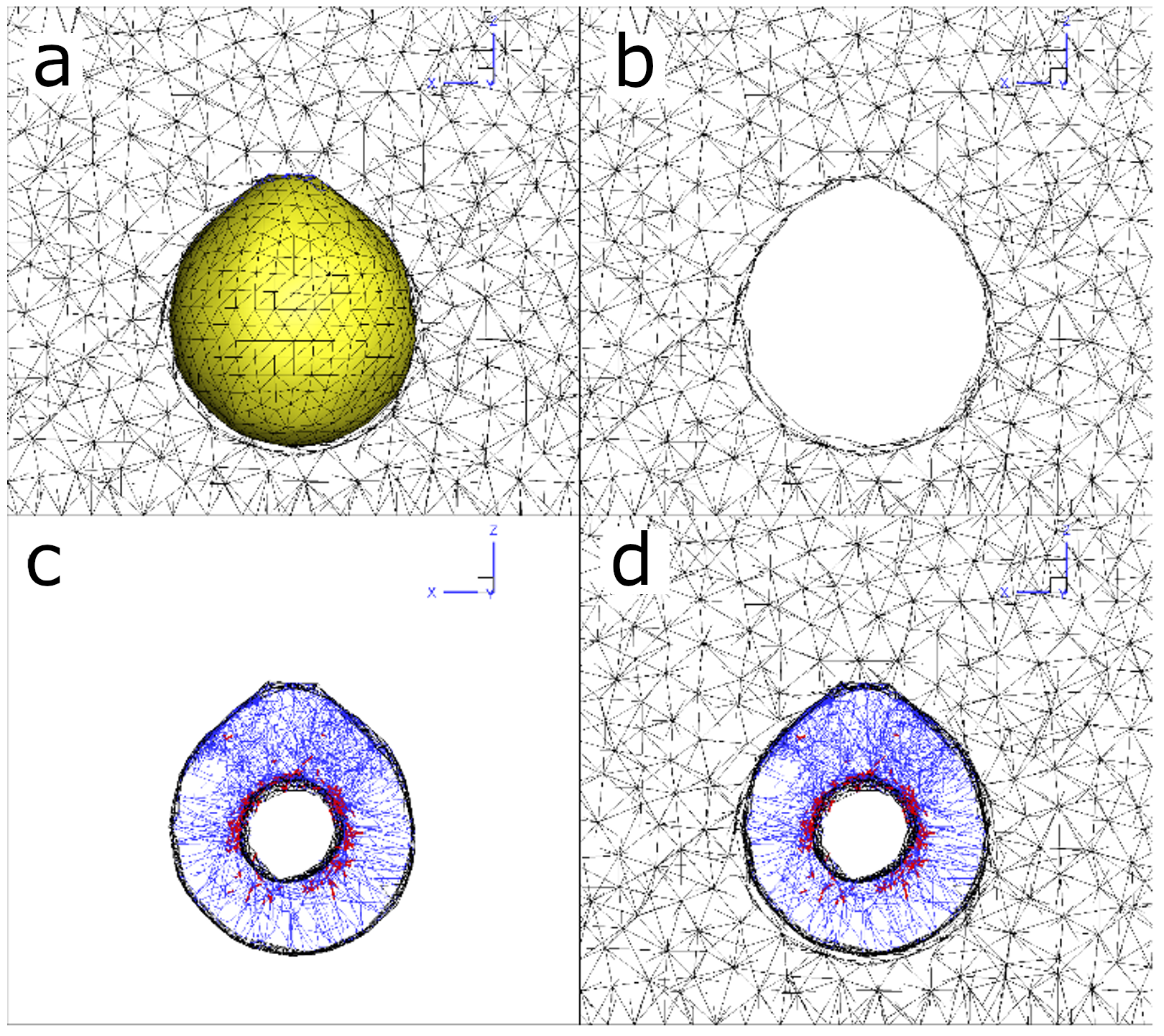


**Supplementary Figure 3**. Volume exclusion effect of the cell. Top view plots of **a**), and **b**) show distributions of ECM fibers with the cell, and without the cell, respectively. Side view plots of **c**), and **d**) show distributions of ECM fibers with the cell, and without the cell, respectively. Note slice thickness of both plots were set to be 5 µm to visualize the volume exclusion effect of the cell clearly.


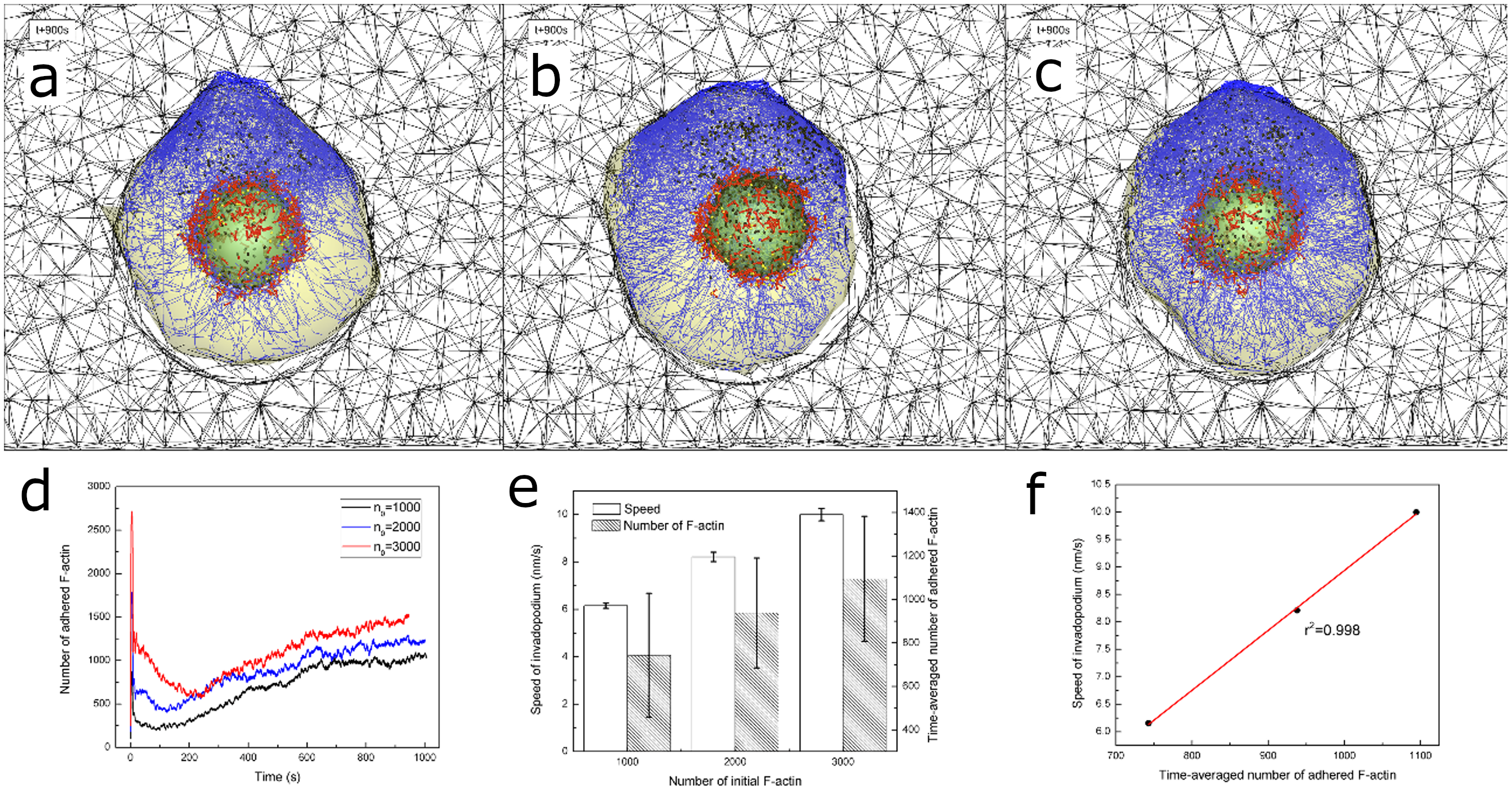


**Supplementary Figure 4**. Effect of initial number of F-actin. Selected still shots of simulated invadopodia protrusion into ECM under three different initial numbers of F-actin, such as **a**) 1000, **b**) 2000, and **c**) 3000. Blue, red, yellow, dark blue, and black line indicate F-actin, bipolar myosin filament, α-actinin, filamin, and fascin, respectively. **d**) A graph showing number of adhered F-actin to the cellular membrane by time. **e**) One bar graph showing simulated speed of invadopodia verses number of initial F-actin, and the other bar graph showing time-averaged number of adhered F-actin verses number of initial F-actin. **f**) Linear regression (r^2^=0.998) between simulated speed of invadopodia and time-averaged number of adhered F-actin in **e**).

**Supplementary Table 1**. List of simulation parameters.

| **Parameter** | **Definition** | **Value** | **Sources** |
| --- | --- | --- | --- |
| *A* | Area [µm^2^] |  |  |
| *A_f_* | Averaged cross-sectional area of a single collagen fiber [$\mathrm{nm}^{2}$] | 1.32×10^3^ |  |
| $A_{A}$ | Average cross-sectional area of the actin filament [$\mathrm{nm}^{2}$] | 38.48 |  |
| *C* | Dissipation energy coefficient [N s m^-1^] |  |  |
| $C_{i,j}^{I}$ | Dissipation energy coefficient of a line element associated with the i-th and j-th nodes on the invadopodial membrane [N s m^-1^] | 0.01~0.02 | C |
| $C_{i,j}^{FTL}$ | Dissipation energy coefficient of a line element associated with the *i*-th and *j*-th nodes on the FTL [N s m^-1^] | 0.01~0.02 | C |
| $C_{i,j}^{ACL}$ | Dissipation energy coefficient of a line element associated with the *i*-th and *j*-th nodes on the ACL mesh [N s m^-1^] | 0.01~0.02 | C |
| $C_{i,j}^{AF}$ | Dissipation energy coefficient of a line element of the *j*-th nodes on the *i*-th actin filament [N s m^-1^] | 0.05~0.07 | C |
| $C_{i,ARP}^{AF}$ | Dissipation energy coefficient of ARP2/3 complex [N s m^-1^] | 0.1 | C |
| $C_{i,j}^{N}$ | Dissipation energy coefficient of a line element with the *i*-th and *j*-th nodes of nuclear membrane [N s m^-1^] | 0.01~0.02 | C |
| $C_{1}^{ACL}$ | Dissipation energy coefficient of actin cortex layer (ACL) which is connected by a line element associated with the *i*-th node on the FTL mesh and the *i*-th node on the ACL mesh [N s m^-1^] | 0.5 | C |
| $C_{1}^{FTL}$ | dissipation energy coefficient of a line element associated with the *i*-th node on the invadopodial membrane and the *i*-th node on the force transduction layer (FTL) [N s m^-1^] | 0.2 | C |
| $C_{0}^{FTL}$ | Viscous drag coefficient of the *i*-th node of FTL [N s m^-1^] | 0.001 | C |
| $C_{0}^{ACL}$ | Viscous drag coefficient of the *i*-th node on the ACL [N s m^-1^] | 0.001 | C |
| $C_{0}^{I}$ | Viscous drag coefficient of a node on the invadopodial membrane [N s m^-1^] | 0.001 | C |
| $C_{0}^{AF}$ | Viscous drag coefficient of the actin filament [N s m^-1^] | 0.001 | C |
| *C_c_* | Friction coefficients associated with the energy dissipation at the integrin node [N s m^-1^] | 0.001 | C |
| *C_cort_* | Drag coefficients associated with viscoelastic behaviors in actin cortex | 0.006 | C |
| *C_e_* | Friction coefficients associated with the energy dissipation at the ECM fiber node [N s m^-1^] | 0.001 | C |
| *C_f_* | Friction coefficients associated with the energy dissipation at the filopodial node [N s m^-1^] | 0.001 | C |
| *C_t_* | Friction coefficients associated with the energy dissipation at the transduce node [N s m^-1^] | 0.001 | C |
| *C_n_* | Friction coefficients associated with the energy dissipation at the nuclear node [N s m^-1^] | 0.001 | C |
| $f_{0}$ | Average polymerization force exerted by single actin filament [pN] | 20 | [1] |
| $f_{cortex}$ | Tension at a node on the ACL [pN] | variable |  |
| ***F*** | Force [N] |  |  |
| $F_{p}$ | Average polymerization force exerted by single actin filament [pN] | 20 | [1] |
| $\boldsymbol{F}_{E,i,j}^{A}$ | Elastic force at the *j*-th node on the *i*-th actin filament [N] |  |  |
| $\boldsymbol{F}_{arp,i,j}^{A}$ | Elastic Arp2/3 force the *j*-th node on the *i*-th actin filament [N] |  |  |
| $\boldsymbol{F}_{br,i,j}^{A}$ | Elastic branch force the *j*-th node on the *i*-th actin filament [N] |  |  |
| $\boldsymbol{F}_{C,i,j}^{A}$ | Contractile force at the *j*-th node on the *i*-th actin filament by a bipolar myosin filament [N] |  |  |
| $\boldsymbol{F}_{L,i,j}^{A}$ | Linking elastic forces at the *j*-th node on the *i*-th actin filament [N] |  |  |
| $\boldsymbol{F}_{P,i}^{C}$ | Polymerization force due to Brownian ratchet motion [nN] | $F_{p}log\left( \frac{v_{0}}{\left\Vert{\hat{\boldsymbol{n}}}_{R,i}^{I}\cdot\left( \boldsymbol{v}_{b}-\boldsymbol{v}_{a} \right) \right\Vert} \right)$ | [2] |
|  |  |  |  |
| $E_{A}$ | Young’s modulus value of actin filament [GPa] | 1.8 | [3] |
| $E_{f}^{e}$ | Young’s modulus value of single collage type 1 fiber [MPa] | 1 | C |
| $k_{on}^{0}$ | Zero forward reaction rate [molecule^−1^ s^−1^] | 1 | C |
| *L* | Length |  |  |
| *L_b_* | Stretched length of bonds between receptors and ligands |  |  |
| $L_{arp,i,j}^{A}$ | Distance between the *j*-th node on the *i*-th actin filament and the first node on the *k*-th actin filament [nm] |  |  |
| $L_{arp,i,j}^{A0}$ | Equilibrium distance between the *j*-th node on the *i*-th actin filament and the first node on the *k*-th actin filament [nm] | 30 | C |
| $L_{i,j}^{A0}$ | Unstressed length of the *j*-th line element of the *i*-th actin filament [nm] | 150 | C |
| $L_{i,j}^{A}$ | Stressed length of the *j*-th line element of the *i*-th actin filament [nm] | variable |  |
| $L_{l}^{A}$ | Stressed length of the *l*-th crosslinking molecule [nm] |  |  |
| $L_{l}^{A0}$ | Unstressed length of the *l*-th crosslinking molecule [nm] | 30 | C |
| $L_{l}^{BMF}$ | Stressed length of the *l*-th bipolar myosin filament [nm] |  |  |
| $L_{l}^{BMF0}$ | Unstressed length of the *l*-th bipolar myosin filament [nm] | 300 | [4] |
| $L_{i,j}^{e}$ | Stressed length of the *j*-th segment of the *i*-th fiber [µm] | variable |  |
| $L_{i,j}^{e0}$ | Unstressed length of the *j*-th segment of the *i*-th fiber [µm] | variable |  |
| *N_e_* | Number of nodes at ECM fiber networks | 30k~234k |  |
| $N_{ACL}$ | Number of nodes on the ACL | 549 |  |
| $N_{I}$ | Number of nodes on the invadopodial membrane | 549 |  |
| $N_{FTL}$ | Number of nodes on the FTL | 549 |  |
| *N_t_* | Number of nodes at transduce layer | 549 |  |
| *N_n_* | Number of nodes at nuclear membrane | 549 |  |
| $n_{i}^{e}$ | Number of nodes at the i-th fiber |  |  |
| $n_{b,i}^{I}$ | Number of integrin-collagen bonds at the *i*-th node of invadopodial membrane |  |  |
| $n_{i}^{A}$ | Number of nodes in i-th actin filament | variable |  |
| $n_{F}$ | Number of actin filaments interacting with the node on the ACL | variable |  |
| ${\hat{\boldsymbol{n}}}_{R,i}^{I}$ | Unit vector normal to the surface of *i*-th invadopodial membrane |  |  |
| *H* | Total elastic energy |  |  |
| $H_{L,i}^{A}$ | Total elastic energy stored in the *i*-th actin filament |  |  |
| $H_{L,l}^{A}$ | Elastic energy at the *l*-th actin-crosslinker |  |  |
| $H_{B,i}^{A}$ | Total elastic energy associated with the change of angle between $L_{i,j}^{A}$and $L_{i,j+1}^{A}$on the *i*-th actin filament |  |  |
| $H_{BR,i,j}^{A}$ | Total branch elastic energy at the *j*-th node on the *i*-th actin filament |  |  |
| $H_{C,l}^{BMF}$ | Elastic energy at the *l*-th bipolar myosin filament, which connect the *j*-th line element on the *i*-th actin filament and the *m*-th line element on the *k*-th actin filament |  |  |
| *h_p_* | Gap between the barbed-end of actin filament and the surface of ACL [nm] |  |  |
| $\kappa$ | Effective spring constant [N/m] |  |  |
| $\kappa_{arp}$ | Effective spring constant of Arp2/3 complex [N/m] | 0.01 | C |
|  |  |  |  |
| $\kappa_{L}^{I}$ | Effective stiffness constant of the line elements of the invadopodial membrane [N/m] | 5.0×10^-5^ | [5] |
| $\kappa_{L}^{FTL}$ | Effective spring constant of line element of the FTL [N/m] | 8.0×10^-3^ | C |
| $\kappa_{A}^{I}$ | Effective stiffness constant of area elements of the invadopodial membrane [ N/m^2^] | 1.0×10^-4^ | C |
| $\kappa_{C}$ | Effective spring constant of line element of the ACL [N/m], | 1.0×10^-2^ | C |
| $\kappa_{T}$ | Effective spring constant of line element of the FTL [N/m] | 8.0×10^-3^ | C |
| $\kappa_{LR}$ | Effective spring constant of ligand-receptor bond [pN/nm] | 1.0 | [6] |
| $\kappa_{b}^{A}$ | Bending modulus of actin filament | $\frac{{\pi E}_{A}r_{A}^{4}}{4}$ |  |
| $\kappa_{br, ang}^{A}$ | Angular bending modulus at the node of Arp2/3 complex [$pN\cdot nm$] | 2690 | [7] |
| $\kappa_{br, dihed}^{A}$ | Dihedral bending modulus at the node of Arp2/3 complex [$pN\cdot nm$] | 2690 | [7] |
| $\kappa_{L,i,j}^{A}$ | Effective stiffness constants of the *j*-th line elements of the *i*-th actin filament [N/m] | $\frac{A_{A}E_{A}}{L_{i,j}^{A0}}$ |  |
| $\kappa_{L}^{A}$ | Effective stiffness constant of the actin-crosslinkers [N/m] | 0.01 | C |
| $\kappa_{C}^{BMF}$ | Effective stiffness constant of the bipolar myosin filament [N/m] | 0.01 | C |
| $\kappa_{f,s}^{e}$ | The stretching modulus of a fiber [nN] | 0.615~1.32 | C |
| $\kappa_{f,b}^{e}$ | The bending modulus of a fiber [pN µm^2^] | (3.02~12.81)×10^-3^ | C |
| $\kappa_{b}^{AF}$ | The bending modulus of a actin filament |  |  |
| $\theta_{i,j}^{A}$ | Stressed angle at the *j*-th node between two segments in the *i*-th actin filament |  |  |
| $\theta_{i,j}^{A0}$ | Unstressed angle at the *j*-th node between two segments in the *i*-th actin filament |  |  |
| $\theta_{i j}^{e}$ | Stressed angle at the *j*-th node between two segments in the *i*-th fiber |  |  |
| $\theta_{i j}^{e0}$ | Unstressed angle at the *j*-th node between two segments in the *i*-th fiber |  |  |
| $\phi_{i,j}^{A}$ | Branched angle between $L_{i,j}^{A}$and $L_{k,1}^{A}$. |  |  |
| $\phi_{i,j}^{A0}$ | Equilibrium branched angle between the mother and daughter filaments | 70˚ |  |
| $\psi_{i,j}^{A}$ | Dihedral angle between two planes, formed by the points $\left(x_{i,j+1}^{A},x_{i,j}^{A},x_{k,1}^{A}\right)$and$\left(x_{i,j}^{A},x_{k,1}^{A},x_{k,2}^{A}\right)$ |  |  |
| $\psi_{i,j}^{A0}$ | Equilibrium dihedral angle between two planes, formed by the points $\left(x_{i,j+1}^{A},x_{i,j}^{A},x_{k,1}^{A}\right)$and$\left(x_{i,j}^{A},x_{k,1}^{A},x_{k,2}^{A}\right)$ | 0˚ |  |
| *k_off_* | Kinetic dissociation rate [s^-1^] |  |  |
| $k_{off}^{0}$ | Kinetic dissociation rate at an unstressed state [s^-1^] | 1 | C |
| $\hat{n}_{w}$ | Unit normal vector at the local surface of the fiber |  |  |
| $r_{A}$ | Radius of actin filament [nm] | 3.5 |  |
| *t* | Time [s] |  |  |
| $\hat{t}_{i,k}$ | Tangential unit vector at the *k*-th segment in the *i*-th fiber |  |  |
| ***v*** | Velocity vector [nm/s] |  |  |
| $v_{a}$ | Velocity vector at the node of actin cortex layer mesh |  |  |
| $v_{b}$ | Velocity vector at the barbed-end of actin filament |  |  |
| $v_{g}$ | Growth rate at the barbed-end of F-actin with 1) nucleate state or ratchet state [nm/s] | 1) 20, and  2) $v_{0}exp\left( -\frac{f_{cortex}}{{n_{F} f}_{0}} \right)$ | [1,8] |
| $v_{s}$ | Shrinkage rate at the pointed-end of F-actin [nm/s] | 10 | [8] |
| $v_{sliding0}$ | The sliding rate of myosin in the absence of load [nm/s] | 160 | [9] |
| ***x*** | Location vector [µm] |  |  |
| $x_{i,1}^{A}$ | Location vector at the pointed-end of *i*-th actin filament |  |  |
| $\boldsymbol{x}_{i,n_{i}^{A}}^{A}$ | Location vector at the barbed-end of *i*-th actin filament |  |  |
| *x_L,i_* | Root of ligand-receptor bonds on the local surface of a fiber [nm] |  |  |
| $x_{ij}^{e}$ | The *j*-th location vector along to the *i*-th fiber [µm] |  |  |
| ***λ*** | Equilibrium distance of an integrin [nm] | 30 | [10] |
| $\lambda_{arp}$ | Equilibrium distance of Arp2/3 complex [nm] | 30 | C |
| **Sup** |  |  |  |
| *A* | Actin |  |  |
| *BMF* | Bipolar myosin filament |  |  |
| *C* | Actin cortex layer (ACL) |  |  |
| *e* | Extracellular matrix |  |  |
| *T* | Force transduce layer |  |  |
| *N* | Nuclear membrane |  |  |
| *I* | Invadopodial membrane |  |  |
| *i* | *i*-th node |  |  |
| *t* | transduce layer |  |  |
| *0* | Previous time or initial state |  |  |
| *1* | Present time |  |  |
| **Sub** |  |  |  |
| *A* | Area |  |  |
| *E* | Elastic |  |  |
| *FC* | Focal complex |  |  |
| *I* | Invadopodial membrane |  |  |
| *L* | Length |  |  |
| *P* | Actin polymerization |  |  |
| *T* | Transduce layer |  |  |
| *b* | bonds |  |  |
| *c* | cytoskeleton |  |  |
| *e* | extracellular matrix |  |  |
| *n* | nucleus |  |  |
| *t* | transduce layer |  |  |

^*^C means “current work”.

**Movie legends for the supplementary movies**

**Supplementary Movie 1.** Simulation of invadopodia protrusion dynamic model into an ECM fiber network over 1000 seconds. An example of simulated invadopodia protrusion into ECM under protrusive duration time of 240 seconds, retractile duration time of 60 seconds, and severing duration time of 20 seconds. Blue, red, yellow, dark blue, and black lines indicate F-actin, bipolar myosin filament, alpha-actinin, fascin and filamin, respectively.

**Supplementary Movie 2.** Simulation of ECM degradation by MMP-2 over 1000 seconds. An example of time-varying contour plots of MMP-2 concentration while simulated invadopodia protrusion into ECM under protrusive duration time of 240 seconds, retractile duration time of 60 seconds, and severing duration time of 20 seconds.

**Supplementary Movie 3.** Experimental observation of cancer cell migration into ECM (control case). Time-lapse images of MDA-MB-231 cancer cell migration into ECM for 1.5 hours (control case).

**Supplementary Movie 4.** Experimental observation of cancer cell migration into ECM (a-MT1-MMP). Time-lapse images of MDA-MB-231 cancer cell migration with blocking antibody against MT1-MMP.

**Supplementary Movie 5.** Simulation of invadopodia protrusion with MT1-MMP knockout and Arp2/3 inhibition. An example of simulated invadopodia protrusion model with MT1-MMP knockout and Arp2/3 inhibition into ECM under protrusive duration time of 240 seconds, retractile duration time of 60 seconds, and severing duration time of 20 seconds. Blue, red, yellow, dark blue, and black lines indicate F-actin, bipolar myosin filament, alpha-actinin, filamin, and fascin, respectively.

**Supplementary Movie 6.** Simulation of invadopodia protrusion with knockout of alpha-actinin over 1000 seconds. An example of simulated invadopodia protrusion model with knockout of alpha-actinin into ECM under protrusive duration time of 240 seconds, retractile duration time of 60 seconds, and severing duration time of 20 seconds. Blue, red, yellow, dark blue, and black lines indicate F-actin, bipolar myosin filament, alpha-actinin, filamin, and fascin, respectively.

**Supplementary Movie 7.** Simulation of invadopodia protrusion with knockout of filamin and fascin over 1000 seconds. An example of simulated invadopodia protrusion model with knockout of filamin and fascin into ECM under protrusive duration time of 240 seconds, retractile duration time of 60 seconds, and severing duration time of 20 seconds. Blue, red, yellow, dark blue, and black lines indicate F-actin, bipolar myosin filament, alpha-actinin, filamin, and fascin, respectively.

**Supplementary Movie 8.** Simulation of invadopodia protrusion during the directed cell migration towards stiffer ECM over 560seconds. An example of simulated invadopodia protrusion during the directed cancer cell migration towards stiffer ECM under protrusive duration time of 300 seconds, retractile duration time of 60 seconds, and severing duration time of 20 seconds. Blue, red, yellow, dark blue, and black lines indicate F-actin, bipolar myosin filament, alpha-actinin, filamin, and fascin, respectively.

**Supplementary Methods**

**Computational model of actin branched network**

**1) Initial number of actin filament and G-actin concentration in reservoir pool**

We assumed that G-actin concentration at the cell front was ~10 µM. The volume of cell with a diameter of 10µm is $5.233\times{10}^{-13}L$. Then, the number of G-actin ($N_{G-actin}$) in a cell can be calculated as $5.233\times{10}^{-15}mole=3.15\times{10}^{6} Gactin$. Since the diameter of G-actin is 7 nm, and the typical length of F-actin is ~ 3 µm, the number of G-actin in a double strand F-actin can be calculated as 857 G-actin. Thus, maximum number of F-actin with the length of 3 µm in a cell can be calculated as ~3677 $\left( \cong{3.15\times{10}^{6} Gactin}/{857 Gactin} \right)$. Thereby, we set 500 actin filaments and 2.72$\times{10}^{6}$ G-actin (reservoir pool) as an initial condition. In case a pointed-end of actin filament depolymerizes, that short actin segment will be removed from the pointed-end and next segment become pointed-end. Then, removed segment will be stored in the reservoir pool, and will be recycled later when polymerization process occurs at the barbed-end of actin filament.

**2) Volume exclusion between ECM fibers and the cellular membrane**

There is no formula for the volume exclusion between actin filaments and cell membrane surfaces. Instead, two conditions were checked when barbed-ends of actin filaments interacted with cell membrane surfaces during the polymerization process: 1) whether barbed-ends of actin filaments were out of actin cortex layer (ACL) or not, and 2) which ACL’s (triangular) elements were closest to barbed-ends of actin filaments. When these two conditions were satisfied, it was assumed that barbed-ends of actin filaments were connected to the ACL’s elements.

**Computational model of a discrete ECM fiber network**

**1) Simulation of discrete ECM fiber mechanics (Module E)**

We assume the ECM fiber network to be composed of viscoelastic ECM fibers and crosslinks, which make strong bonds between adjacent fibers [11]. The elastic energy stored in the ECM fiber network can be expressed in terms of the stretching and bending properties of the constituent fibers. The stretching modulus of a fiber is given by $\kappa_{f,s}^{E}\left(=E_{f}^{E}A_{f}\right)$, where $E_{f}$and $A_{f}\left(=\pi r_{f}^{2}\right)$ are the Young’s modulus (1 MPa) and the cross-sectional area of a single fiber, respectively. The bending modulus of a fiber is given by$\kappa_{f,b}^{E}\left(=E_{f}I_{f}\right)$, where [12]. The stretching elastic energy of the *j*-th segment of the *i*-th fiber is given as a function of the difference between the stressed ($L_{i j}^{e}$) and unstressed ($L_{i j}^{e0}$) lengths, and the bending elastic energy as the one of stressed ($\theta_{i j}^{E}$) and unstressed ($\theta_{i j}^{E0}$) angles at the *j*-th node between two segments in the *i*-th fiber. The total elastic energy in the *i*-th ECM fiber in the network can be expressed as following:

$H_{f,i}^{E}=\frac{\kappa_{f,s}^{E}}{2}\sum_{j=1}^{N_{i}^{E}} \frac{\left(L_{i j}^{E}-L_{i j}^{E0}\right)^{2}}{L_{i j}^{E0}}+\frac{\kappa_{f,b}^{E}}{2}\sum_{j=1}^{N_{i}^{E}} \frac{\left(\theta_{i j}^{E}-\theta_{i j}^{E0}\right)^{2}}{L_{i j}^{E0}}.$ (S1)

Here, it should be noted that the elastic energy at the *j*-th node in the *i*-th fiber is summed only for coaxial neighbouring nodes. Similarly, the elastic force at the *j*-th node in the *i*-th fiber,$F_{E,i j}^{E}$, can be derived by using the virtual work theory:

$F_{E,i j}^{E}=-\frac{\partial H_{f,i}^{E}}{\partial x_{i j}^{E}}=-\kappa_{f,s}^{E}\sum_{k=j}^{j+1} \frac{\left(L_{i k}^{E}-L_{i k}^{E0}\right)}{L_{i k}^{E0}}\frac{\partial L_{i k}^{E}}{\partial x_{i j}^{E}}-\kappa_{f,b}^{E}\sum_{k=j-1}^{j+1} \frac{(\theta_{i k}^{E}-\theta_{i k}^{E0})}{L_{i k}^{E0}}\frac{\partial\theta_{i k}^{E}}{\partial x_{i j}^{E}}$ (S2)

where $\theta_{i k}^{E}=\cos^{-1}\left(\hat{t}_{i k}\cdot\hat{t}_{i k+1}\right),$ $\hat{t}_{i k}$and $\hat{t}_{i k+1}$are tangential unit vectors at the *k* and *k*+1-st nodes in the *i*-th fiber, respectively, and $\frac{\partial\theta_{ i k}^{E}}{\partial x_{i j}^{E}}=\frac{-1}{\sqrt{1-\left(\hat{t}_{i k}\cdot\hat{t}_{i k+1}\right)^{2}}}\left(\frac{\partial\hat{t}_{i k}}{\partial x_{i j}^{E}}\cdot\hat{t}_{i k+1}+\hat{t}_{i k}\cdot\frac{\partial\hat{t}_{i k+1}}{\partial x_{i j}^{E}}\right).$ To incorporate viscoelastic behaviors in the ECM fiber network, line elements of ECM fibers can be modeled using Kelvin-Voigt model (a spring and a dashpot together in parallel). The dissipation force at the *j*-th node in the *i*-th ECM fiber,$F_{D,i j}^{E0}$, can be expressed as

(S4)

where $C_{i j}^{E}$is coefficient of dissipation energy at the *j*-th node in the *i*-th ECM fiber, respectively. $v_{i j-1}^{E0}$and $v_{i j+1}^{E0}$are velocity vectors at the *j*-*1*th and *j+1th* neighboring nodes at the previous time-step, respectively. To solve the dynamics of ECM fiber network, a dynamic equation at the *j*-th node in the *i*-th ECM fiber can be expressed as

$\left( 2C_{ij}^{E}+C_{0}^{E} \right)\frac{dx_{ij}^{E}}{\mathrm{dt}}=F_{E,ij}^{E}$+$F_{FC,ij}^{E}+F_{D,ij}^{E0}$ (S5)

where $C_{0}^{E}$is a coefficient of self-dissipation energy for the *j*-th node in the *i*-th ECM fiber. Note that dynamics of ECM fibers is explicitly solved using at the previous time-step. $F_{FC,i j}^{E,i}$is a focal complex (FC) force at the j-th node in the *i*-th ECM fiber. Note that dynamics of ECM fibers is coupled with invadopodia protrusion dynamics through an equation of .

We used a very regular structured ECM fiber network which represent a soft ECM gel. In our previous publication, we already characterized three ECM fiber network models with three different pore sizes of 0.5, 1.0 and 1.5 µm and three different fiber diameters of 28, 34 and 41 nm, respectively (16). First, we aimed to simulate mechanical stretching tests for the three ECM fiber network models in order to characterize and compare the three models with experimentally measured bulk moduli of ECM gels. Various simulations of mechanical stretching tests for each ECM fiber network model were performed using two parameters of single fiber diameters and moduli. We found that the moduli of the model are linearly and significantly increased as single fiber diameter or single fiber modulus is increased in each ECM model. Both simulation and experiment show an excellent agreement over both ECM fiber diameters of 28, 34 and 41 nm and ECM pore sizes of 0.5, 1.0 and 1.5 μm. Second, we predicted 3D cell-ECM interactions and penetration speeds by comparing with experimental observations. We have found that speeds of both tip and root of filopodia increase as the pore size is increased in experiments. The simulated speeds of both tip and root of filopodia, too, shows a trend similar to the experiment. The simulations and experiment have shown an excellent agreement for all the ECM pore sizes of 0.5, 1.0 and 1.5 um (or pH levels of 9, 7 and 5).

**2) Simulation of reaction diffusion mass transfer (Module RD)**

To consider chemical interactions of the ECM fiber network with a cancer cell, we model the degradation, proteolysis, and haptotaxis of the ECM fiber network. Six reaction-diffusion equations for concentrations of MMP-2 (), TIMP-2 (), MT1-MMP (), a ternary complex of MT1-MMP:TIMP-2:proMMP-2 () [13], ligands () (or collagen molecules) and ECM () are numerically solved using Finite Volume Method (FVM) [14]. Constitutive partial differential equations for the six biochemical concentrations are summarised in followings (see Supplementary Fig. 1):

(S6)

$\frac{\partial\phi_{2}}{\partial t}=\nabla\cdot\left( D_{\phi_{2}}\nabla\phi_{2} \right)-k_{\phi_{1}:\phi_{2}}^{on}\phi_{1}\phi_{2}-k_{\phi_{2}:\phi_{3}}^{on}\phi_{2}\phi_{3}+k_{\phi_{4}}^{off}\phi_{4}+\alpha_{\phi_{2}}\left( x_{tip}^{I} \right)\phi_{5}$ (S7)

$\frac{\partial\phi_{3}}{\partial t}=-k_{\phi_{2}:\phi_{3}}^{on}\phi_{2}\phi_{3}+k_{\phi_{4}}^{off}\phi_{4}-k_{\phi_{3}}^{decay}\phi_{3}+\alpha_{\phi_{3}}\left( x_{tip}^{I} \right)\phi_{5}$ (S8)

(S9)

(S10)

(S11)

whereand are decay coefficients of MMP-2 (0.0017 s^-1^), and ligands (0.0001 s^-1^), respectively. is a degradation coefficient of ECM (1.04×10^6^ M^-1^s^-1^). is a kinetic association rate constant for binding TIMP-2 with MMP2 (5×10^5^ M^-1^s^-1^) and its term physically represents the reduction of MMP-2 by the endogenous soluble inhibitor TIMP-2. is a kinetic association rate constant for binding the ternary complex with MT1-MMP (1.95×10^4^ M^-1^s^-1^), which results in the release of activated MMP-2. is a kinetic association rate constant for binding TIMP-2 with MT1-MMP (2.74×10^6^ M^-1^s^-1^), and is a kinetic dissociation rate constant of the ternary complex for unbinding TIMP2 and MT1-MMP (2×10^-4^ s^-1^). and represent secretion rates of TIMP2 (1.0×10^-3^ M s^-1^) and MT1-MMP (1.0×10^-1^ M s^-1^) at the tip of invadopodium, respectively. In particular, $x_{tip}^{I}$ indicates the tip of invadopodia, and MT1-MMP and TIMP-2 secretions at the invadopodial membrane are modelled as source terms [15]. The last term of Eq. S8, $\alpha_{\phi_{3}}\left( x_{tip}^{I} \right)\phi_{5}$, indicate a linear function of ligand density with slope of $\alpha_{\phi_{3}}\left( x_{tip}^{I} \right)$. The ligand densities of soft and stiff ECM were calculated as 3.3 and 8.3 µM based on collagen densities of 1 mg/ml (soft) and 2.5 mg/ml (stiff), and the molecular weight of collagen is ~300000 g/mol.

To incorporate the degradability factor into the ECM fiber network and its nonlinear behavior under mechanical responses, we consider that each crosslink node comprises crosslink molecules, such as amino acids, that can rupture. We model the degradability of ECM fiber network by considering detachment events among the *i*-th crosslink node and its neighboring fibers, and the degradability of ECM fiber network depends on a local value of the ECM integrity ($I_{ECM,i}={\phi_{6,i}}/{\phi_{6,i}^{0}};0\leq I_{ECM,i}\leq1$). Here, $\phi_{6,i}$ and $\phi_{6,i}^{0}$ are concentrations of the *i*-th ECM node at present and initial states (10μM), respectively. The number of uncrosslinked (or degraded) fibers at the i-th crosslink node,$N_{i}^{uf}$, is calculated as $N_{i}^{uf}=\left( 1-I_{ECM,i} \right)N_{i}^{f0}$, where $N_{i}^{f0}$ is an initial number of crosslinked ECM fibers at the i-th crosslinks node.

**Supplementary References**

1. Upadhyaya, A., Chabot. J. R., Andreeva, A., Samadani, A. & van Oudenaarden, A. Probing polymerization forces by using actin-propelled lipid vesicles. *Proc. Natl Acad. Sci. USA.* **100**, 4521-4526 (2003).
2. Mogilner, A. & Oster, G. Force generation by actin polymerization II: the elastic ratchet and tethered filaments. *Biophys. J.* **84**, 1591-1605 (2003).
3. Kojuma, H., Ishjima, A. & Yanagida, T. Direct measurement of stiffness of single actin filaments with and without tropomyosin by in vitro nanomanipulation. *Proc. Natl. Acad. Sci. USA*. **91**, 12962-12966 (1994).
4. Melli, L. *et al*. Bipolar filaments of human nonmuscle myosin 2-A and 2-B have distinct motile and mechanical properties. *eLife* **7**, 332871 (2018).
5. Drury, J. L. & Dembo, M. Aspiration of human neutrophils: Effects of shear thinning and cortical dissipation. *Biophy. J.* **81**, 3166–3177 (2010).
6. Dembo, M. On peeling an adherent cell from a surface. In: Vol. 24 of series: Lectures on Mathematics in the Life Sciences, Some Mathematical problem in Biology. Providence: American Mathematical Society. 51–77 (1994).
7. Popov, K., Komianos, J. & Papoian, G. A. MEDYAN: Mechanochemical Simulations of Contraction and Polarity Alignment in Actomyosin Networks. *PLoS Comput. Biol.* **12**, e1004877 (2016).
8. Zhu, J. & Mogilner, A. Mesoscopic model of actin-based propulsion. *PLoS Comput. Biol*. **8**, e1002764 (2012).
9. Erdmann, T., Albert, P. J. & Schwarz, U. S. Stochastic dynamics of small ensembles of non-processive molecular motors: The parallel cluster model. *J. of Chem. Phys*. **139**, 175104 (2013).
10. Kanchanawong, P. *et al*. Nanoscale architecture of integrin-based cell adhesions. *Nature* **468**, 580–586 (2010).
11. Stein, A. M., Vader, D. A., Weitz, D. A. & Sander, L. M. The micromechanics of three-dimensional collagen-I gels. *Complexity* **16**, 22–28 (2011).
12. Yang, Y. *et al*. (2008) Mechanical properties of native and cross-linked type i collagen fibrils. *Biophys. J.* **94**, 2204–2211.
13. Deakin, N. E. & Chaplain, M. A. J. Mathematical modeling of cancer invasion: the role of membrane-bound matrix metalloproteinases. *Frontiers in Oncology* **3**, 1–9 (2013).
14. Kim, C. J. Kim A coordinate-free form of the finite gradient in discretizing scalar and momentum diffusion. In proceedings of the 4^th^ JSME-KSME Thermal Engineering Conference, Kobe, Japan (2000).
15. Poincloux, R., Lizárraga, F. & Chavrier, P. Matrix invasion by tumour cells: a focus on MT1-MMP trafficking to invadopodia. *J. Cell Sci.* **122**, 3015–3024 (2009).
16. Kim, M.-C., Whisler, J., Silberberg, Y. R. , Kamm, R. D. & Asada, H. H. Cell invasion dynamics into a three dimensional extracellular matrix fibre network. PLoS Compu. Biol. 11, e1004535 (2015).
